# LINC00022 acts as an oncogene in colorectal cancer progression via sponging miR-375-3p to regulate FOXF1 expression

**DOI:** 10.1186/s12885-022-09566-5

**Published:** 2022-04-26

**Authors:** Lingling Xu, Hongmei He, Yu Shang, Xiaona Qu, Jinghua Sun

**Affiliations:** Department of Gastrointestinal Oncology, The Second Hospital of Dalian Medical University, No. 467 Zhongshan Road, Dalian, 116027 Liaoning China

**Keywords:** Colorectal cancer, lncRNA LINC00022, miR-375-3p, FOXF1

## Abstract

**Background:**

Abnormal expression of long non-coding RNAs (lncRNAs) has been shown to be associated with the pathogenesis of cancers, including colorectal cancer (CRC). It has been reported that LINC00022 is highly expressed in some typs of cancer and its overexpression indicates poor prognosis. The function of LINC00022 in CRC progression remains unclear and is mainly investigated in the present study.

**Methods:**

LINC00022 expression in CRC tissues was analyzed by using the TNMplot software. LINC00022 expression in CRC cells was measured by quantitative real-time PCR. The effects of LINC00022 on the malignant behaviors of CRC cells were detected by a series of in vitro and in vivo experiments. Dual-luciferase assays were used to verify the targeting relationship between LINC00022 and miR-375-3p and between miR-375-3p and Forkhead box F1 (FOXF1), followed by the rescue experiment.

**Results:**

LINC00022 was highly expressed in CRC tissues compared with paired para-carcinoma tissues (*n* = 41). CRC cells with LINC00022 knockdown exhibited decreased cell proliferation, migration, and invasion abilities but increased apoptosis accompanied by decreased protein levels of c-Myc, cyclin D1, cleaved caspase 3, cleaved poly(ADP-ribose) polymerase, matrix metalloproteinase (MMP) 2, and MMP9. Additionally, LINC00022 downregulation in CRC cells suppressed the tube formation of human umbilical vein endothelial cells (HUVECs) as evidenced by decreased vascular endothelial growth factor A levels in LINC00022-silenced cells. The inhibitory effect of LINC00022 knockdown on tumor growth was also observed in an in vivo model. Conversely, LINC00022 overexpression showed that opposite effect. We further demonsrtaed that LINC00022 could upregulate FOXF1 expression through sponging miR-375-3p. Moreover, miR-375-3p knockdown reversed the effects of LINC00022 down-regulation.

**Conclusions:**

LINC00022 may up-regulate FOXF1 expression via competitively binding miR-375-3p, thereby promoting the development of CRC.

**Supplementary Information:**

The online version contains supplementary material available at 10.1186/s12885-022-09566-5.

## Background

Colorectal cancer (CRC) is the third most common malignancy in the world, with more than 2 million new cases and more than 1 million deaths each year [1, 2]. In recent years, with the development of diagnosis and chemoradiotherapy, the mortality rate of CRC patients has declined [3, 4]. However, there is currently no curative treatment, and patients with CRC are often diagnosed at an advanced stage [5, 6]. The prognosis of patients with advanced CRC is poor and the long-term survival rate after surgery is very low [7]. Therefore, exploring the pathogenesis and potential therapeutic targets of CRC may have important implications for the treatment of CRC.

Long non-coding RNAs (lncRNAs) is defined as RNA molecules with more than 200 nucleotides and no protein-coding potential [8]. LncRNAs are identified to regulate gene expression through a series of mechanisms, including transcription, post-transcriptional processing, genome imprinting, and chromatin modification [9]. It has been reported that lncRNAs are associated with multiple physiological and pathological processes, including tumors [10]. Many lncRNAs are dysregulated and play a promoting or suppressing role in the development of cancer [11]. Specifically, LINC00022 expression was significantly up-regulated in non-small cell lung cancer (NSCLC) tissues, and LINC00022 down-regulation inhibited the tumorigenesis in NSCLC cells [12]. Overexpression of LINC00022 promoted the proliferation, migration, and invasion abilities of esophageal cancer cells but reduced apoptosis [13]. Meanwhile, a tumor suppressor role of LINC00022 is also observed in some types of cancer. For instance, LINC00022 was down-regulated in laryngeal cancer tissues, and the up-regulation of LINC00022 significantly reduced cell proliferation, migration, and invasion in laryngeal cancer cells [14]. These observations suggest that LINC00022 functions differently in different types of cancer. However, the expression and function of LINC00022 in CRC are currently unknown.

LncRNAs are usually reported to regulate mRNA expression via acting as a sponge [15]. LINC00022 is also reported to regulate cancer development through endogenous RNA (ceRNA) networks in some types of cancers. For example, LINC00022 promoted the malignant behaviors of esophageal cancer cells by up-regulating the expression of E2F transcription factor 7 through competitively binding to miR-30e-5p [13]. LINC00022 accelerated the development of NSCLC through sponging miR-30a-5p [12]. However, whether LINC00022 participates in CRC progression through the ceRNA network needs further investigation. In the present study, we mainly explored the expression, function, and potential regulatory mechanism of LINC00022 in CRC to provide a theoretical basis for the treatment of CRC.

## Methods

### Cell culture and transfection

CRC cell lines (DLD1, HCT116, CaCo-2, SW480, HCT-15, and RKO) were obtained from Procell Life Science&Technology Co,.Ltd. (Wuhan, China). HCT116 cells were cultured in McCoy’s 5A medium (NO. PM150710; Procell, Wuhan, China). CaCo-2 and RKO cells were cultured in Minimum Eagle's Medium (NO. 41500–067; Gibco, Grand Island, NY, USA). DLD1, SW480, and HCT-15 cells were cultured in Roswell Park Memorial Institute (RPMI)-1640 medium (NO. R1383; Sigma-Aldrich, St.Louis, MO, USA). All the culture media contained 10% fetal bovine serum (FBS; NO. B548117; Sangon Biotech, Shanghai, China) and cell cultures in respective culture media were placed in an incubator at 37℃ with 5% CO_2_. To up-regulate LINC00022 expression, the sequence of LINC00022 was cloned into a lentiviral expression vector, named Lv-LINC00022. To silence LINC00022 expression, short hairpin RNA (shRNA)-1 and shRNA-2 specifically targeting LINC00022 were cloned into a lentiviral vector named Lv-anti-LINC00022-1 and Lv-anti-LINC00022-2. Cells were infected with lentivirus for 72 h and then used in subsequent experiments.

### Quantitative real-time PCR (qRT-PCR)

TRIpure Reagent (NO. RP1001; BioTeke, Beijing, China) was used to extract total RNA from tissues and cell lines. RNA was then reversely transcribed into complementary DNA by using Super M-MLV reverse transcriptase (NO. PR6502; BioTeke) and RNase inhibitor (NO. RP5602; BioTeke), and qRT-PCR was then carried out using 2 × Power Taq PCR MasterMix (NO. PR1702; BioTeke), SYBR Green (NO. S9430; Sigma-Aldrich). β-actin served as an internal control for LINC00022, Forkhead box F1 (FOXF1), and vascular endothelial growth factor A (VEGFA). 5S RNA served as an internal control for miR-375-3p. Data were analyzed using the 2 − ^△△ct^ method. The primers used in the present study were:

LINC00022 F, 5’- AGAATGAAGACCACAAT-3’;

R, 5’- CTTGGTAAAAGGAGAAT-3’.

FOXF1 F, 5’- CATCCAGAGTTCACCCACCAA-3’;

R, 5’- CGCCTGGCATTTCCTTCG-3’.

VEGFA F, 5’- TCACCAAGGCCAGCACATAG-3’;

R, 5’- GGGCACCAACGTACACGCT-3’.

miR-375-3p F, 5’-TTTGTTCGTTCGGCTCGCGTGA-3’;

R, 5’-GCAGGGTCCGAGGTATTC-3’.

### CCK-8 assay

Cell viability was detected using the CCK-8 assay. Cells were seeded in 96-well plates at a density of 4 × 10^3^ cells per well. After infected with lentivirus for 0, 24, 48, 72, and 96 h, respectively, cells at each time point were treated with CCK-8 solution (10 μl; NO. 96992; Sigma-Aldrich) and cultured in an incubator for 1 h at 37℃ with 5% CO_2_. The optical density (OD) at 450 nm was detected by using the microplate reader (NO. ELX-800; Biotek, Winooski, VT, USA).

### Cell cycle and apoptosis assays

Cell cycle distribution was measured by using a cell cycle kit (NO. KGA512; KeyGEN, Nanjing, China). Briefly, the lentivirus-infected cells were treated with RNaseA (100 μl) and incubated in a water bath at 37℃ for 30 min, and then treated with propidium iodide (PI) staining solution (400 μl) at 4℃ for 30 min in the dark. Apoptosis was detected by using an apoptosis detection kit (NO. KGA106; KeyGEN). In brief, the infected cells were treated with Annexin V-FITC (5 μl) and PI (5 μl) for 15 min at room temperature in the dark. The apoptosis rate and cell cycle distribution were detected by the flow cytometry (NO. NovoCyte; Aceabio, San Diego, CA, USA).

### Western blot

Cell lysis buffer for Western and IP (NO. P0013; Beyotime) mixed with phenylmethyl sulfonylfluoride (NO. ST506; Beyotime) was used to extract total proteins from cells and tissues. An equal amount of proteins were loaded in each lane and separated with SDS-PAGE gel and then transferred to polyvinylidene difluoride membranes. The membranes were incubated with primary antibodies overnight at 4℃. After being incubated with corresponding secondary antibody (NO. A0208 and A0216; Beyotime, Shanghai, China) for 2 h at room temperature, the proteins were visualized by enhanced chemiluminescence system reagent (NO. P0018; Beyotime). The β-actin served as an internal control. The primary antibodies used for Western blot were shown as follows: c-Myc (1:1000; NO. A1309; ABclonal, Wuhan, China), cyclin D1 (1:1000; NO. A19038; ABclonal), caspase 3 (1:1000; NO. A19654; ABclonal), poly(ADP-ribose) polymerase (PARP; 1:1000; NO. A19596; ABclonal), matrix metalloproteinase 2 (MMP2; 1:1000; NO. 10373–2-AP; Proteintech, Wuhan, China), matrix metalloproteinase 9 (MMP9; 1:1000; NO. 10375–2-AP; Proteintech), the vascular endothelial growth factor A (VEGFA; 1:1000; NO. DF7470; Affinity, Cincinnati, OH, USA), FOXF1 (1:1000; NO. A03563-1; Boster, Wuhan, China), Signal transducer and activator of transcription 3 (STAT3; 1000; NO. AF6294; Affinity), p-STAT3 (1:1000; NO. AF3293; Affinity), and β-actin (1:1000; NO. sc-47778; Santa Cruz Biotechnology, Santa Cruz, CA, USA).

### Wound-healing assay

Cell migration was assessed by wound-healing assay. After 72 h of lentivirus infection, the scratch test was performed. The cells (4 × 10^5^ cells/well) in each group were scratched with a 200 μl pipette tip, and the surface of the cells was washed with serum-free medium to remove the cell debris. After lentivirus infection, the cells were observed under a microscope (NO. IX53; Olympus, Tokyo, Japan; 100 ×) and photographed. The cell positions in the photos were recorded for subsequent photography. Cells were cultured in an incubator at 37℃ with 5% CO_2_ for 24 h and then photographed. The migration distance of each experimental group was calculated.

### Transwell assay

The transwell assay was performed to evaluate cell invasion. The Transwell chamber (NO. 3422; Corning, NY, USA) coated with Matrigel was placed in a 24-well plate, and culture medium containing 30% FBS was added in the lower chamber (800 μl). Cell suspension (200 μl) was added to the upper chamber, and the cell number was 2 × 10^4^ per well. 24-well plates were cultured in an incubator for 24 h under the conditions of 37℃, 5% CO_2_, and saturated humidity. The Transwell chamber was fixed at room temperature with 4% paraformaldehyde for 15 min and stained with 0.4% crystal violet solution for 5 min. The invaded cells were counted under an inverted microscope (200 × ; NO. IX53; Olympus).

### HUVEC-related tube formation assay

Human umbilical vein endothelial cell (HUVEC) suspension (100 μl) was inoculated into 96-well plates coated with Matrigel gel, and the number of cells was 1 × 10^4^ per well. The cells were cultured in an incubator at 37℃ for 18 h, and the formation of tubes was observed and photographed under an inverted phase-contrast microscope (100 × , Olympus).

### Dual-luciferase assay

The binding between LINC00022 and miR-375-3p was predicted by LncBase Predicted v.2 (http://carolina.imis.athena-innovation.gr/diana_tools/web/index.php?r=lncbasev2%2Findex-predicted). The binding between miR-375-3p and the 3’-untranslated regions (UTR) of FOXF1 was analyzed by TargetScan Human 7.2 (http://www.targetscan.org/vert_72/).

The LINC00022 sequence with wild-type (wt) or mutant type (mut) miRNA binding sites was cloned into the luciferase reporter vector. The 3’-UTR of FOXF1 with wt or mut miRNA binding sites was cloned into the luciferase reporter vector. The recombinant luciferase reporter vector containing wt-LINC00022, wt-3’-UTR of FOXF1 or the corresponding mut sequences was co-transfected with miR-375-3p mimics or NC mimics into 293 T cells. Forty-eight hours after co-transfection, luciferase activity was detected by a luciferase assay kit (NO. E1910; Promega, Madison, WI, USA). Renilla luciferase activity was normalized to firefly luciferase activity.

### Tumor xenografts

All the animal experiments were performed according to the National Institutes of Health Guide for the care and use of laboratory animals and approved by the ethic committee of Dalian Medical University (NO. 2020–001, 2020–3-26). Nude mice aged 5–6 weeks were randomly divided into 4 groups after 1 week of adaptive feeding, namely: LV-anti-NC, Lv-anti-LINC00022, Lv-NC, and Lv-LINC00022. CRC cells (1 × 10^6^) with stable low LINC00022 expression or stable LINC00022 overexpression were injected into the subcutaneous tissue of nude mice. Tumor formation was observed after injection, and tumor tissue diameter and volume were measured every 3 days. After 22 days, all mice were anesthetized by intraperitoneal injection of pentobarbital sodium (150 mg/kg) and tumor tissues were collected and photographed.

### Immunohistochemistry

Tumor tissues were embedded in paraffin and sectioned (5 μm) and then rehydrated with a series of graded ethanol dilutions. The slices were placed in a hot antigen repair solution and heated in a microwave oven for 5 min for antigen retrieval. After treated with 3% H_2_O_2_ at room temperature for 15 min, the sections were incubated with goat serum at room temperature for 15 min. Then, the slides were incubated with primary antibody (FOXF1; 1:50; NO. D160340-0025; Sangon Biotech, Shanghai, China or Ki67; 1:100; NO. WL01384a; Wanleibio, Shenyang, China) overnight at 4℃, followed by the incubation of horseradish peroxidase (HRP)-labeled secondary antibody (1:500; NO. #31,460; Thermo Fisher Scientific, Waltham, MA, USA) at room temperature for 2 h. After developed in 3,3-diaminobenzidine tetrahydrochloride and counterstained with hematoxylin, the sections were observed and photographed under a microscope.

### Statistical analysis

Statistical analysis was conducted using GraphPad Prism 8 software. The t-test, one-way ANOVA or two-way ANOVA was used to evaluate the difference between different groups. Because the sample sizes were small (N ≤ 6), effect sizes were calculated for all samples to confirm the power of the analysis [16]. Literature has shown that t-tests are acceptable for small sample sizes when the effect size is large (effect size > 0.8) [17, 18]. In the present study, the effect sizes analyzed by G*Power 3 software were all > 0.8. All data were presented as the mean ± standard deviation (SD). *P* < 0.05 was statistically significant.

## Results

### LINC00022 was highly expressed in CRC tissues

**Fig. 1:**
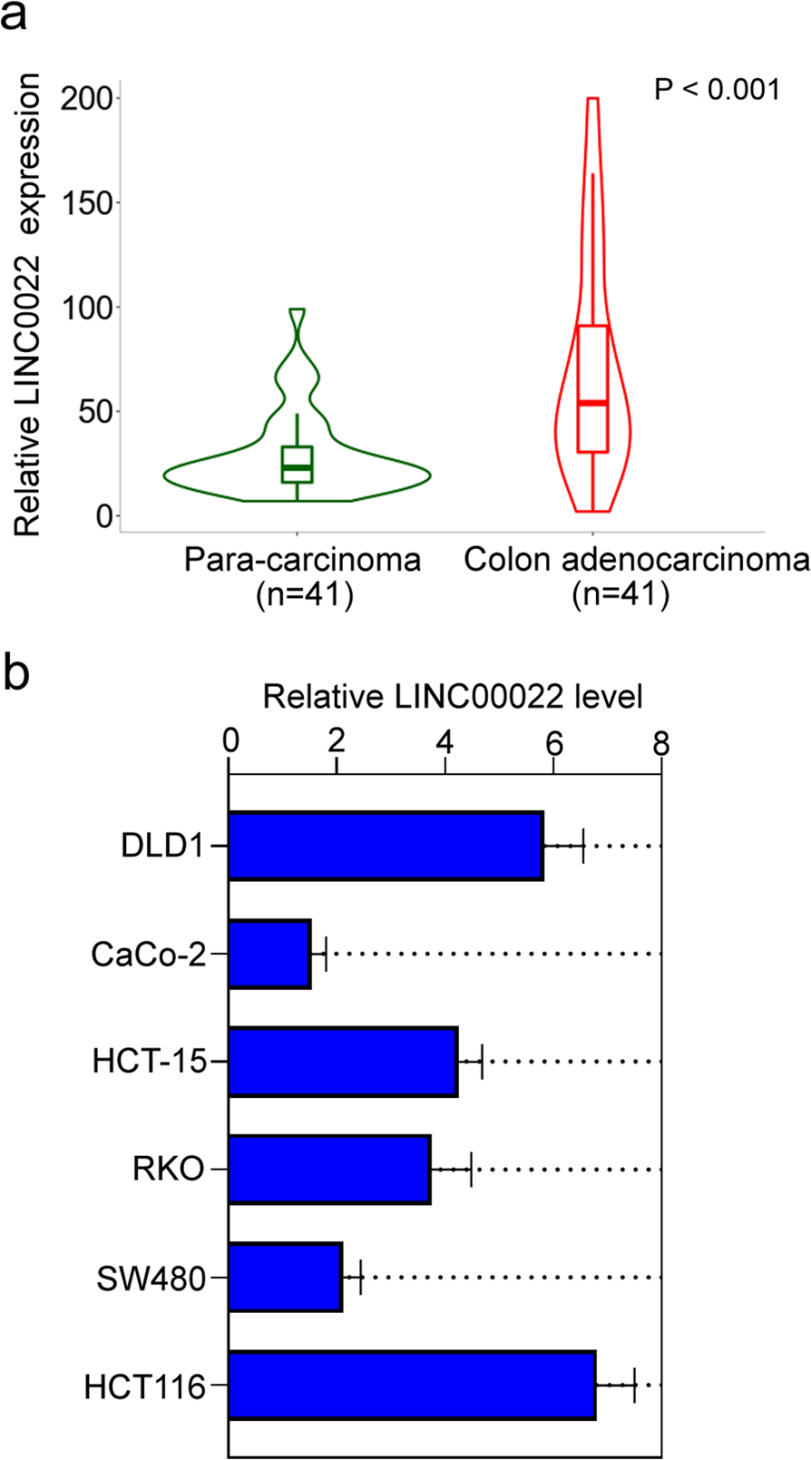
LINC00022 was highly expressed in CRC tissues. LINC00022 expression in CRC tissues and paired normal tissues (*n* = 41) was analyzed by TNMplot software based on he Cancer Genome Atlas (TCGA) data (**a**). The mRNA expression levels of LINC00022 in CRC cells (DLD1, CaCo-2, HCT-15, RKO, SW480, and HCT116) were measured by qRT-PCR (**b**). Data were presented as mean ± standard deviation (SD). *N* = 3. CRC, colorectal cancer; qRT-PCR, quantitative Real-time PCR

The expression of LINC00022 in CRC tissues was first explored by analyzing The Cancer Genome Atlas (TCGA) data using the TNMplot software (https://tnmplot.com/analysis/) [19]. The results showed that LINC00022 was significantly increased in CRC tissues (*n* = 41) compared with paired para-carcinoma tissues (Fig. 1a). qRT-PCR was performed to measure LINC00022 expression in CRC cell lines (DLD1, CaCo-2, HCT-15, RKO, SW480, and HCT116). HCT116 and DLD1 cell lines with relatively higher LINC00022 expression and CaCo-2 cell line with relatively lower LINC00022 expression were selected for subsequent experiments (Fig. 1b).

### LINC00022 induced proliferation and inhibited apoptosis in CRC cells

**Fig. 2:**
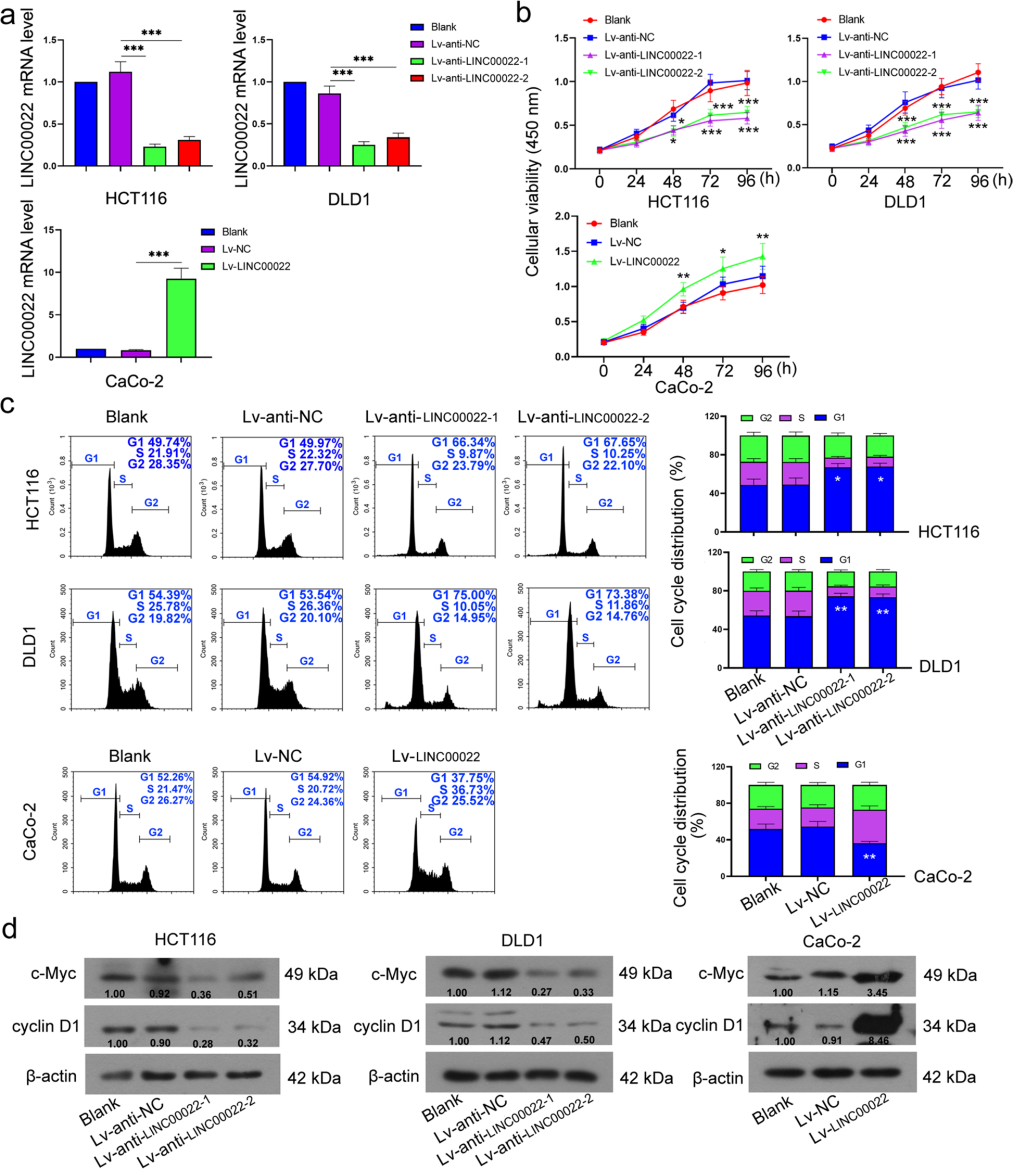
LINC00022 promoted proliferation in CRC cells. After HCT116, DLD1, and CaCo-2 cells were infected with LINC00022 low expression lentivirus or high expression lentivirus, relative mRNA expression levels of LINC00022 in CRC cells were detected by qRT-PCR (**a**); The viability of HCT116, DLD1, and CaCo-2 cells were measured using CCK-8 assay (**b**); Cell cycle distribution of HCT116, DLD1, and CaCo-2 cells was detected by flow cytometry (**c**); Relative protein expression levels of c-Myc and cyclin D1 were assessed by Western blot (**d**); β-actin served as internal control. Data were presented as mean ± standard deviation (SD). *N* = 3. *P < 0.05, **P < 0.01 and ***P < 0.001 vs Lv-anti-NC group or Lv-NC group. NC, negative control

**Fig. 3:**
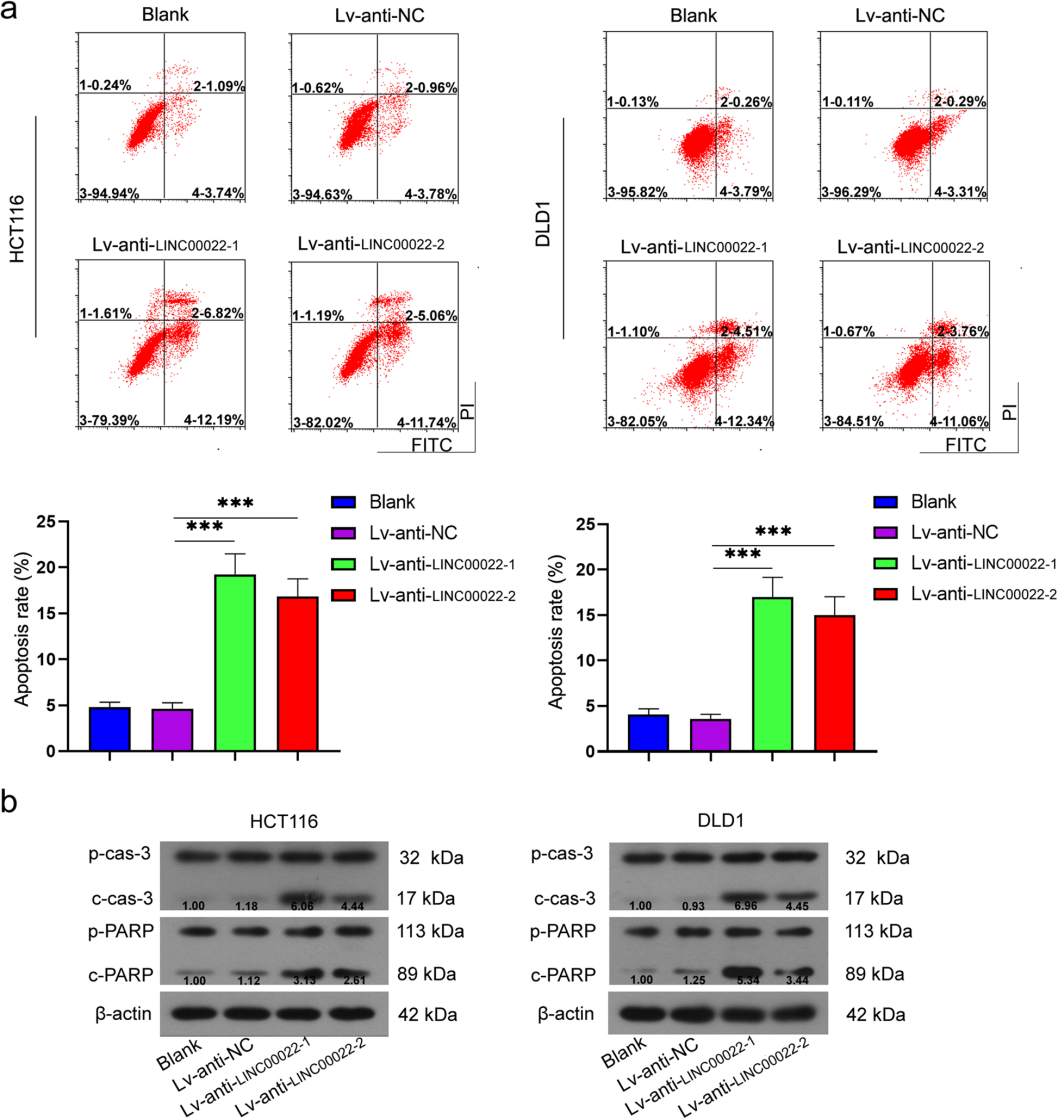
LINC00022 inhibited apoptosis CRC cells. After HCT116, DLD1, and CaCo-2 cells were infected with LINC00022 low expression lentivirus, the apoptosis of HCT116 and DLD1 cells was detected by flow cytometry (**a**); The protein expression levels of pro caspase 3, cleaved caspase 3, pro PARP, and cleaved PARP were measured using Western blot (**b**). β-actin served as internal control. Data were presented as mean ± standard deviation (SD). *N* = 3. ***P < 0.001 vs Lv-anti-NC group or Lv-NC group. PARP, poly(ADP-ribose) polymerase

To further explore the function of LINC00022 in CRC, we knocked down or overexpressed the levels of LINC00022 in CRC cells. The knockdown and overexpression efficiencies of LINC00022 in HCT116 (0.21–0.28 fold vs Lv-anti-NC group), DLD1 (0.3–0.4 fold vs Lv-anti-NC group), and CaCo-1 (11.2 fold vs Lv-NC group) cells were detected by qRT-PCR (Fig. 2a). Results of the CCK-8 assay showed that LINC00022 overexpression obviously increased the viability of CRC cells compared with the Lv-NC group (1.3–1.4 fold), whereas LINC00022 silencing decreased cell viability (0.6–0.7 fold) compared with the Lv-anti-NC group (Fig. 2b). Meanwhile, LINC00022 overexpression significantly decreased number of cells in the G1 phase (0.7 fold vs Lv-NC group), while LINC00022 knockdown (1.4 fold vs Lv-anti-NC group) showed the opposite result (Fig. 2c). Additionally, the protein expression levels of c-Myc and cyclin D1 in CRC cells were significantly increased by LINC00022 overexpression and decreased by LINC00022 downregulation (Fig. 2d). Conversely, the apoptosis rate was significantly increased by LINC00022 downregulation (3.6–4.7 fold vs Lv-anti-NC group, Fig. 3a). Meanwhile, apoptosis-related factors, cleaved caspase 3 and cleaved PARP, were significantly increased in LINC00022-silenced cells (Fig. 3b). Overall, the results suggest that LINC00022 promotes proliferation and suppresses apoptosis in CRC cells.

### LINC00022 promoted migration and invasion in CRC cells

**Fig. 4:**
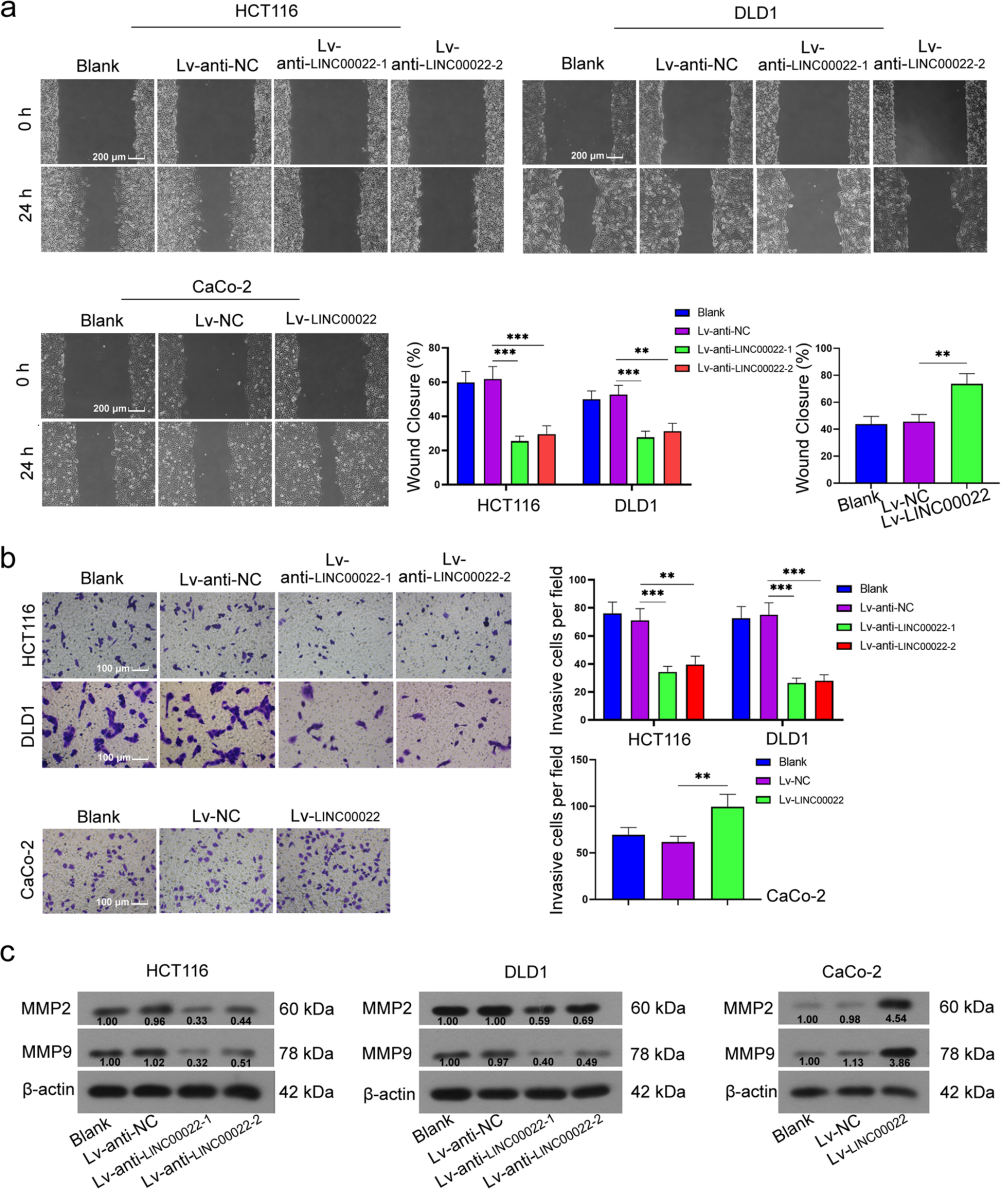
LINC00022 promoted migration and invasion in CRC cells. After HCT116, DLD1, and CaCo-2 cells were infected with LINC00022 low expression lentivirus or high expression lentivirus, the migration (Scale bar = 200 μm) and invasion (Scale bar = 100 μm) in HCT116, DLD1, and CaCo-2 cells were measured using Wound-healing assay and Transwell assay (**a** and **b**); Relative protein expression levels of MMP2 and MMP9 were assessed using Western blot (**c**). β-actin served as internal control. Data were presented as mean ± standard deviation (SD). *N* = 3. **P < 0.01 and ***P < 0.001 vs Lv-anti-NC group or Lv-NC group. MMP2, matrix metalloproteinase 2; MMP9, matrix metalloproteinase 9

We next assessed the effects of LINC00022 on the migration and invasion in CRC cells. It was observed that the migration and invasion abilities of CRC cells was increased by LINC00022 overexpression (1.6 fold vs Lv-NC group), while LINC00022 knockdown had the opposite effects (0.4–0.6 fold vs Lv-anti-NC group, Fig. 4a and 4b). Meanwhile, the results of Western blot assay illustrated that relative protein levels of MMP2 and MMP9 were significantly increased in LINC00022-overexpressed cells, whereas LINC00022 downregulation decreased their levels (Fig. 4c). Taken together, the observations indicate that LINC00022 promotes the migration and invasion of CRC cells.

### LINC00022 promoted angiogenesis in CRC

**Fig. 5:**
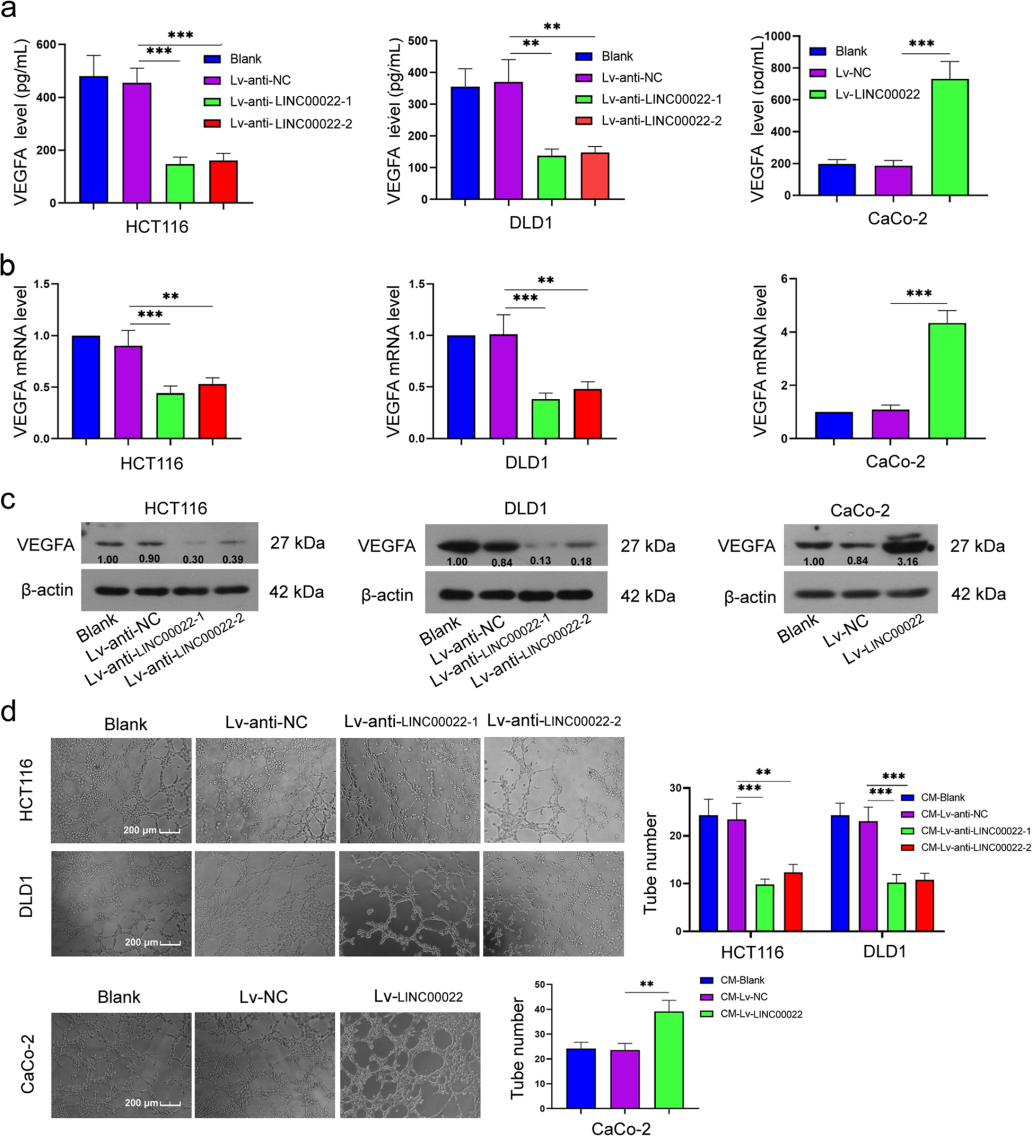
LINC00022 promoted angiogenesis in CRC. After HCT116, DLD1, and CaCo-2 cells were infected with LINC00022 low expression lentivirus or high expression lentivirus, the level of VEGFA in the cell supernatant was detected by the detection kit (**a**); The mRNA and protein levels of VEGFA in HCT116, DLD1, and CaCo-2 cells were measured using qRT-PCR and Western blot, respectively (**b** and **c**); The number of cavity formation was calculated (**d**). Scale bar = 200 μm. Data were presented as mean ± standard deviation (SD). *N* = 3. **P < 0.01 and ***P < 0.001 vs Lv-anti-NC group or Lv-NC group. VEGFA, vascular endothelial growth factor A; qRT-PCR, quantitative Real-time PCR

As shown in Fig. 5a, LINC00022 overexpression significantly increased the content of VEGFA in the cell supernatant (4.0 fold vs Lv-NC group), while LINC00022 knockdown had the opposite effect (0.3–0.4 fold vs Lv-anti-NC group). Similarly, the mRNA (4.0 fold vs Lv-NC group) and protein expression levels of VEGFA in CRC cells were significantly increased by LINC00022 upregulation, while LINC00022 silencing decreased their levels (mRNA, 0.4–0.6 fold vs Lv-anti-NC group, Fig. 5b and 5c). Additionally, LINC00022 upregulation increased HUVECs tube formation (1.7 fold vs Lv-NC group), whereas LINC00022 inhibition reduced HUVECs tube formation (0.4–0.5 fold vs Lv-anti-NC group, Fig. 5d). The data suggest that LINC00022 promotes angiogenesis in CRC.

### LINC00022 upregulated FOXF1 expression through sponging miR-375-3p

**Fig. 6:**
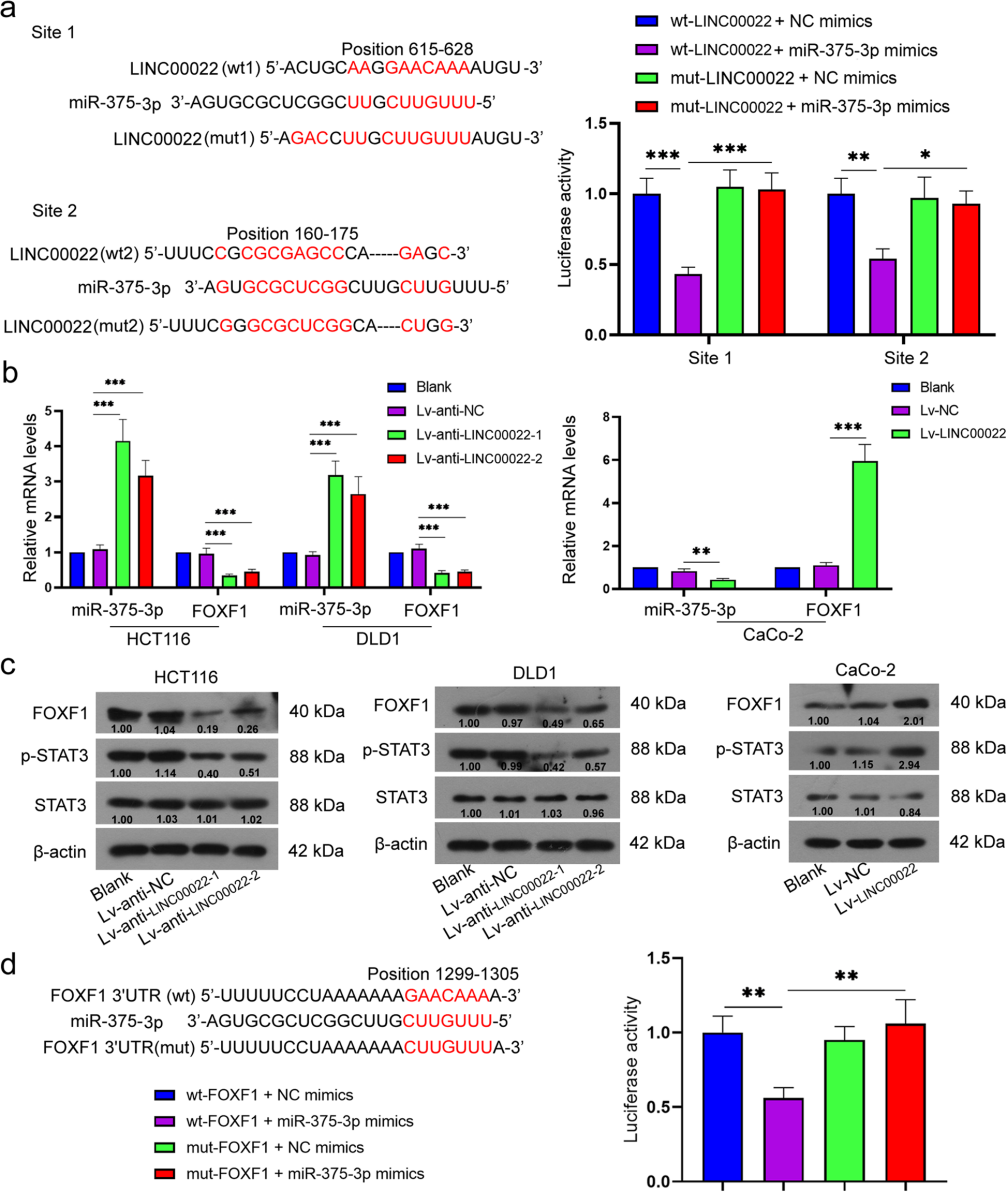
LINC00022 upregulated FOXF1 expression through sponging miR-375-3p. (**a**) The binding sites between LINC00022 and miR-375-3p were predicted by LncBase v.2 and verified by dual luciferase assay. *P < 0.05, **P < 0.01 and ***P < 0.001 vs wt-LINC00022 + miR-375-3p mimics group. After HCT116, DLD1, and CaCo-2 cells were infected with LINC00022 low expression lentivirus or high expression lentivirus, the relative mRNA levels of miR-375-3p and FOXF1 were assessed by qRT-PCR (**b**); **P < 0.01, and ***P < 0.001 vs Lv-anti-NC group or Lv-NC group. Relative protein levels of FOXF1, STAT3, and p-STAT3 were calculated using Western blot (**c**). β-actin served as the internal control. (**d**) The binding sites between miR-375-3p and FOXF1 were predicted by TargetScanHuman 7.2 and verified by dual luciferase assay. **P < 0.01 vs wt-FOXF1 + miR-375-3p mimics group. Data were presented as mean ± standard deviation (SD). *N* = 3. FOXF1, Forkhead Box F1; STAT3, signal transducer and activator of transcription 3, and qRT-PCR, quantitative Real-time PCR

To investigate whether LINC00022 regulates gene expression through a ceRNA mechanism, the bioinformatics website LncBase v.2 predicted that there were potential binding sites between LINC00022 and miR-375-3p (Fig. 6a). To verify the relationship between LINC00022 and miR-375-3p, the dual-luciferase reporter assay was performed. As presented in Fig. 6a, the luciferase activity in wt-LINC00022 + miR-375-3p mimics group was significantly decreased (0.4–0.6 fold) compared with wt-LINC00022 + NC mimics group and mut-LINC00022 + miR-375-3p mimics group, respectively, while no significant changes were observed in other groups. Furthermore, LINC00022 overexpression significantly decreased the expression of miR-375-3p (0.5 fold vs Lv-NC group) and increased FOXF1 (5.4 fold vs Lv-NC group) mRNA level in CRC cells, whereas LINC00022 knockdown had the opposite effect (miR-375-3p, 3.0–4.0 fold; FOXF1, 0.4–0.5 fold vs Lv-anti-NC group, Fig. 6b). Additionally, the results of Western blot showed that the protein levels of FOXF1 and p-STAT3 (Tyr 705) were significantly increased by LINC00022 upregulation, while LINC00022 silencing had the opposite effect (Fig. 6c). The relationship between miR-375-3p and FOXF1 was detected by dual-luciferase reporter assay, and the results showed that the luciferase activity in wt-FOXF1 + miR-375-3p mimics group was relatively weaker (0.5–0.6 fold) compared with that in other groups (Fig. 6d). Moreover, results of Pearson correlation analysis showed that the expression of LINC00022 in CRC tissues was negatively correlated with miR-375-3p and positively correlated with FOXF1 (Supplementary Fig. 1a). miR-375-3p expression was negatively associated with FOXF1 in CRC tissues (SupplementaryFig. 1a). Taken together, these data suggest that LINC00022 may increase FOXF1 expression through sponging miR-375-3p.

### miR-375-3p knockdown reversed the effects of LINC00022 down-regulation in CRC

**Fig. 7:**
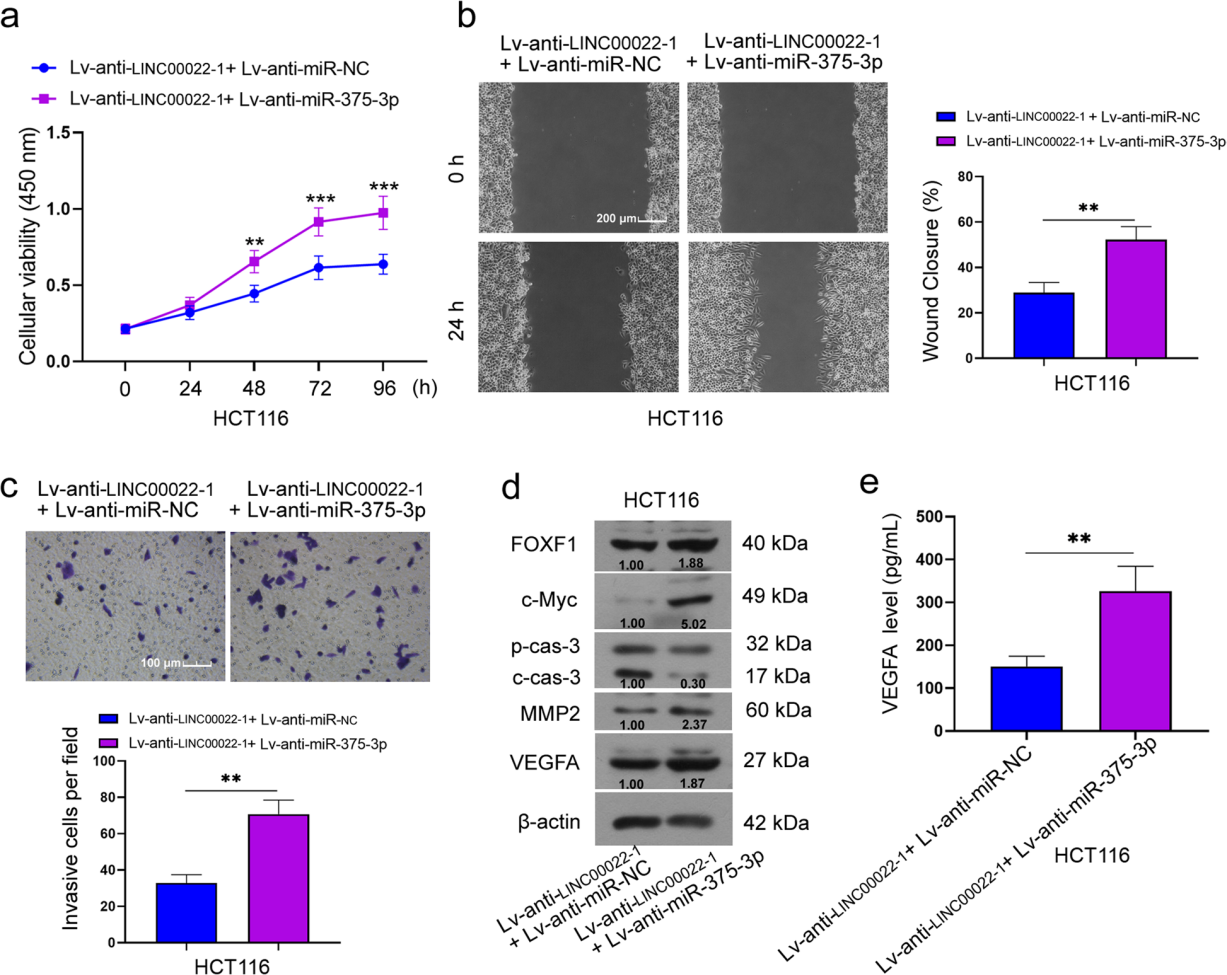
miR-375-3p knockdown reversed the effects of LINC00022 down-regulation on cell function in CRC. After LINC00022-silenced HCT116 cells were infected with Lv-anti-miR-375-3p mimic or Lv-anti-miR-NC, the viability of HCT116 cells was measured by CCK-8 assay (**a**); The migration (Scale bar = 200 μm) and invasion (Scale bar = 100 μm) of HCT116 cells were measured using Wound-healing assay and Transwell assay, respectively (**b** and **c**); Relative protein levels of FOXF1, c-Myc, pro caspase 3, cleaved caspase 3, MMP2, and VEGFA were assessed by Western blot (**d**). β-actin served as internal control. The content of VEGFA in cell supernatant was measured by detection kit (**e**). Data were presented as mean ± standard deviation (SD). N = 3. **P < 0.01 vs Lv-anti-LINC00022 + Lv-anti-miR-NC group. NC, negative control; FOXF1, Forkhead Box F1; MMP2, matrix metalloproteinase 2; VEGFA, vascular endothelial growth factor-A

To explore whether LINC00022 is involved in the development of CRC by regulating miR-375-3p, a series of rescue experiments were performed. Results of the CCK-8 assay displayed that miR-375-3p silencing reversed the decreased cell viability caused by LINC00022 knockdown (1.2–1.5 fold vs Lv-anti-LINC00022-1 + Lv-anti-miR-NC group, Fig. 7a). Furthermore, the migration and invasion in LINC00022-silenced cells were also increased by miR-375-3p downregulation (1.6 fold vs Lv-anti-LINC00022-1 + Lv-anti-miR-NC group, Fig. 7b and 7c). Meanwhile, it was observed that miR-375-3p knockdown dramatically increased the protein expression levels of FOXF1, c-Myc, MMP2, and VEGFA in LINC00022-silenced cells, whereas decreased cleaved caspase 3 protein level (Fig. 7d). The VEGFA content in LINC00022-silenced cells was also increased by miR-375-3p downregulation (2.2 fold vs Lv-anti-LINC00022-1 + Lv-anti-miR-NC group, Fig. 7e). Overall, the results indicate that LINC00022 participates in the development of CRC via regulating the expression of miR-375-3p.

### *LINC00022 promoted the tumor growth of CRC cells *in vivo

**Fig. 8:**
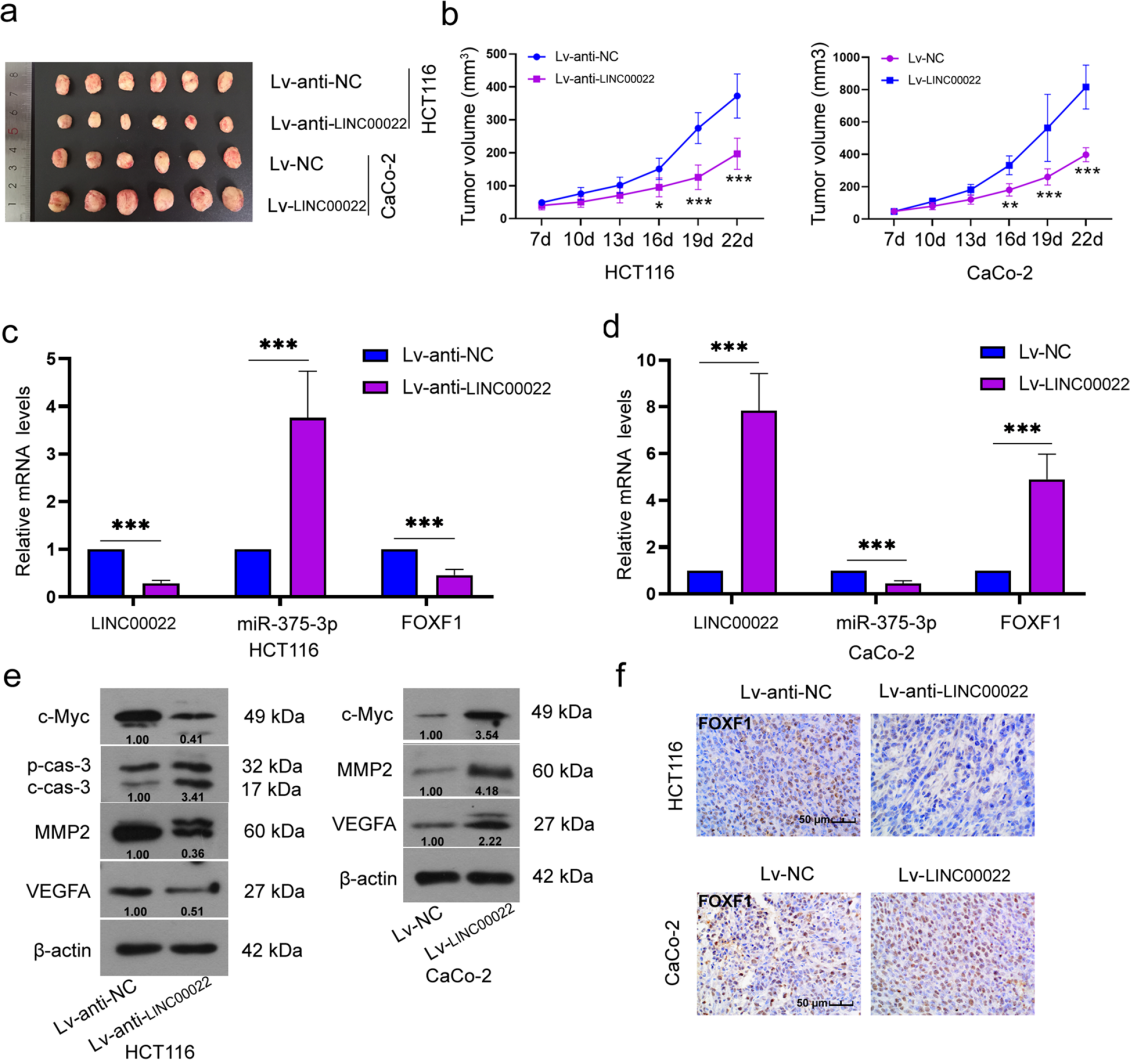
LINC00022 promoted the development of CRC in vivo. HCT116 cells with stable low expression of LINC00022 or CaCo-2 cells with stable overexpression of LINC00022 were injected subcutaneously into nude mice. (**a**) Representative images of tumor tissues in nude mice. (**b**) The volumes were calculated after being injected with LINC00022-silenced cells or -overexpressed cells. (**c** and **d**) The mRNA levels of LINC00022, miR-375-3p, and FOXF1 were detected in tumor tissues. (**e**) Relative protein levels of c-Myc, pro caspase 3, cleaved caspase 3, MMP2, and VEGFA in tumor tissues. (**f**) Immunohistochemical staining of FOXF1 in tumor tissues. Scale bar = 50 μm. β-actin served as the internal control. Data were presented as mean ± standard deviation (SD). *N* = 6. *P < 0.05, **P < 0.01, and ***P < 0.001 vs Lv-anti-NC group or Lv-NC group. FOXF1, Forkhead Box F1; MMP2, matrix metalloproteinase 2; VEGFA, vascular endothelial growth factor-A

To explore the effect of LINC00022 on tumor growth, CRC cells with stable low expression or stable overexpression of LINC00022 were injected subcutaneously into nude mice. The results showed that LINC00022 overexpression increased tumor volume in mice (1.3–2.2 fold vs Lv-NC group), whereas LINC00022 downregulation had the opposite effect (0.5–0.7 fold vs Lv-anti-NC group, Fig. 8a and 8b). It was shown that the mRNA expression levels of LINC00022 and FOXF1 in tumor tissues were significantly increased by the injection of LINC00022 overexpressing cells (4.9–7.8 fold vs Lv-NC group) and decreased by the injection of LINC00022 low-expressing cells (0.3–0.5 fold vs Lv-anti-NC group, Fig. 8c). Conversely, the expression level of miR-375-3p in tumor tissues was significantly decreased (0.5 fold vs Lv-NC group) by LINC00022 overexpression and increased (3.8 fold vs Lv-anti-NC group) by LINC00022 downregulation (Fig. 8d). Additionally, LINC00022 knockdown decreased the protein expression levels of c-Myc, MMP2, and VEGFA in tumor tissues and increased cleaved caspase 3 levels, whereas LINC00022 overexpression increased c-Myc, MMP2, and VEGFA protein levels (Fig. 8e). Immunohistochemistry also confirmed the expression of FOXF1 in tumor tissues (Fig. 8f). Meanwhile, results of immunohistochemistry showed that LINC00022 silencing decreased Ki67 (a typical marker of cell proliferation) expression in tumor tissues, whereas LINC00022 overexpression increased Ki67 expression (Supplementary Fig. 1b). These data indicate that LINC00022 promotes tumor growth.

## Discussion

**Fig. 9:**
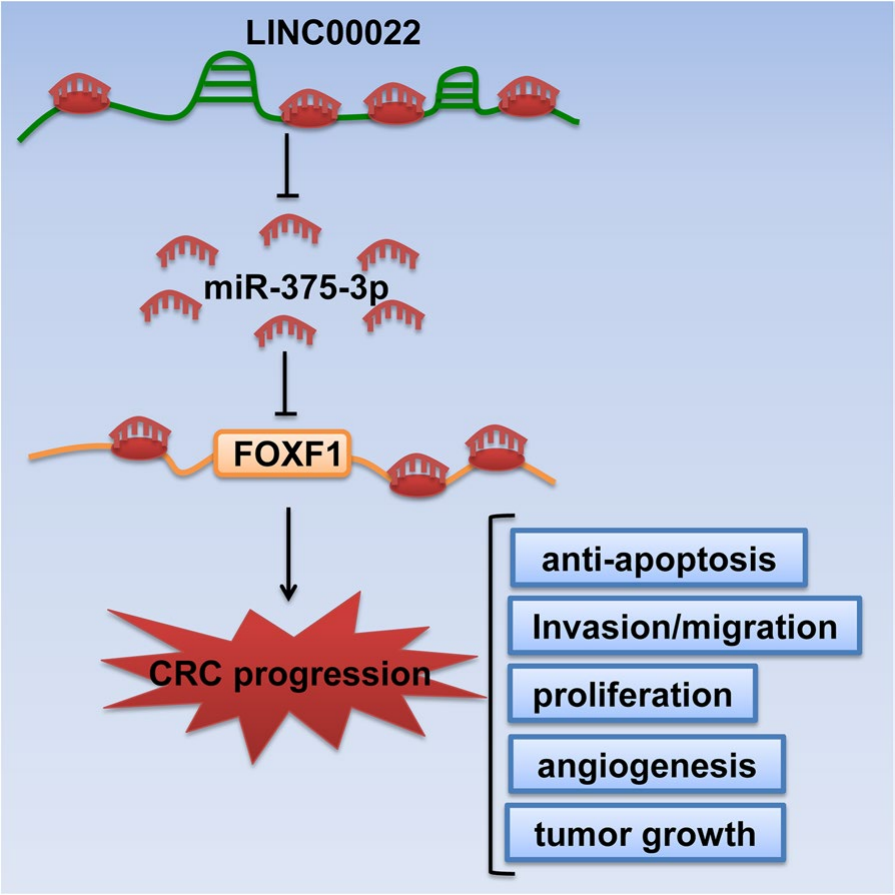
The graphical abstract of this study. LINC00022 may up-regulate FOXF1 expression via competitively binding miR-375-3p, thereby promoting the development of CRC

In the current study, we demonstrated that LINC00022 was highly expressed in CRC tissues compared with paired normal tissues, and LINC00022 knockdown decreased CRC cell viability, migration, invasion, and angiogenesis but inducing apoptosis. Furthermore, we proved that LINC00022 might up-regulate FOXF1 expression through sponging miR-375-3p. Moreover, miR-375-3p knockdown reversed the effects of LINC00022 silencing on the function of CRC cells. Altogether, our results indicate that LINC00022 may up-regulate FOXF1 expression via competitively binding miR-375-3p, thereby promoting the development of CRC (Fig. 9).

LINC00022 was found to be dysregulated in a variety of cancers, including laryngeal squamous cell carcinoma [20], glioma [21], and pancreatic cancer [22]. We found that LINC00022 was highly expressed in CRC tissues, indicating that LINC00022 may be positively associated with the development of CRC. Previous studies have shown that the function of LINC00022 in different types of cancer is contradictory [14, 22]. Here, in vivo and in vitro studies showed that the up-regulation of LINC00022 promoted the proliferation, migration, and invasion of CRC cells and inhibited apoptosis, indicating that LINC00022 plays a promoting role in CRC progression. Additionally, we found that LINC00022 was positively correlated with VEGFA expression and promoted the ability of HUVEC to form capillary-like structures. VEGFA, also known as VEGF, is a member of the VEGF family [23]. VEGFA is a key regulator of pathologic angiogenesis [24]. VEGFA acts as the main mediator of tumor angiogenesis, stimulates the growth of new blood vessels in nearby capillaries, and allows tumors to obtain oxygen and nutrients needed for growth [25]. VEGFA was reported to be highly expressed in CRC and to promote angiogenesis in CRC [26]. Our results suggest that LINC00022 promotes angiogenesis in CRC. Taken together, we demonstrate that LINC00022 promotes the development of CRC by promoting cell proliferation, migration, invasion, and angiogenesis and inhibiting apoptosis.

Many lncRNAs are identified to be involved in the development of cancer by competitively inhibiting miRNA expression. miR-375-3p was considered to be an inhibitor of a variety of cancers, including laryngeal squamous cell carcinoma [27], bladder cancer [28], and gastric cancer [29]. Additionally, it has been previously reported that miR-375-3p was significantly decreased in CRC tissues and cells, and inhibited tumorigenesis and chemoresistance of CRC [30, 31], indicating that miR-375-3p as a suppressor of CRC development may be a potential target for colorectal therapy. The binding between LINC00022 and miR-375-3p was predicted by bioinformatics website and their regulatory relationship was detected by dual-luciferase assay. Moreover, the down-regulation of miR-375-3p reversed the effect of down-regulation of LINC00022 on cell function, indicating that the promoting effect of LINC00022 on the development of CRC may be achieved by competitive inhibition of miR-375-3p. Our findings suggest that the LINC00022/miR-375-3p axis may be one of the pathways that LINC00022 promotes CRC development.

miRNAs are considered to suppress the translation of cognate mRNAs and further regulating the expression of protein-coding genes. FOXF1 belongs to the family of winged-helix transcription factors and has been reported to be involved in the development of many types of cancer. Early studies indicated that FOXF1 promoted the development of cancers, including prostate cancer [32], osteosarcoma [33], and breast cancer [34]. In addition, the FOXF1 expression level was significantly increased in CRC, and FOXF1 overexpression promoted the epithelial-mesenchymal transition, angiogenesis, and chemoresistance of CRC [35, 36], suggesting that FOXF1 may be an oncogene in CRC and is selected as downstream of miR-375-3p. Herein, we confirmed that miR-375-3p could target FOXF1 and negatively regulated its expression. We demonstrated that LINC00022 was positively correlated with FOXF1 and negatively correlated with miR-375-3p, indicating that LINC00022 may promote CRC progression by competitively inhibiting miR-375-3p to upregulate FOXF1 expression. The miR-375-3p/FOXF1 axis may be one of the pathways that LINC00022 promotes CRC development. It is undeniable that LINC00022 as an lncRNA may also regulate the expression of FOXF1 through other mechanisms, both directly binding and indirect regulation need to be further explored in the future. Furthermore, we found that LINC00022 was positively correlated with the expression of STAT3 and p-STAT3 (Tyr 705). Studies have shown that there was an interaction between STAT3 and FOXF1 protein, and FOXF1 mutation inhibited the expression of STAT3 [37]. STAT3 acted as an oncogene in CRC and promoted the growth, invasion, and angiogenesis of CRC [38–40]. The results suggested that LINC00022 may promote the development of CRC in whole or in part by increasing FOXF1-mediated STAT3 expression.

## Conclusions

We demonstrate that LINC00022 promotes the development of CRC via increasing cell viability, migration, invasion, and angiogenesis and inhibits apoptosis. The newly identified LINC00022/miR-375-3p/FOXF1 axis provides novel insight into the development of CRC and may be a potential therapeutic target for the treatment of CRC.

## Supplementary Information

**Additional file 1: Supplementary figure 1. **(a) The expression correlation betweenLLINC0022 and miR-375-3p, FOXP1 in CRC tissues was analyzed by quantitativereal-time PCR. (b) Immunohistochemical staining of Ki67 in tumor tissues. Scale bar = 50 μm.

**Additional file 2: Supplementary figure S2d. **The original blot images of Fig. 2d.

**Additional file 3: Supplementary figure S3b. **The original blot images of Fig. 3b.

**Additional file 4: Supplementary figure S4c. **The original blot images of Fig. 4c.

**Additional file 5: Supplementary figure S5c. **The original blot images of Fig. 5c.

**Additional file 6: Supplementary figure S6c. **The original blot images of Fig. 6c.

**Additional file 7: Supplementary figure S7d. **The original blot images of Fig. 7d.

**Additional file 8: Supplementary figure S8e. **The original blot images of Fig. 8e.

## Data Availability

The datasets used and/or analysed during the current study are available from the corresponding author on reasonable request.

## Abbreviations

LncRNAs: Long non-coding RNAs
CRC: Colorectal Cancer
FOXF1: Forkhead box F1
PARP: Poly(ADP-ribose) polymerase
MMP2: Matrix Metalloproteinase 2
HUVEC: Human Umbilical Vein Endothelial Cell
STAT3: Signal Transducer And Activator Of Transcription 3
