# Supplementary figures and images for: LINC00022 acts as an oncogene in colorectal cancer progression via sponging miR-375-3p to regulate FOXF1 expression
Source: BMC Cancer. 2022 Apr 26;22:453. doi: 10.1186/s12885-022-09566-5 (PMC9040237; doi:10.1186/s12885-022-09566-5)

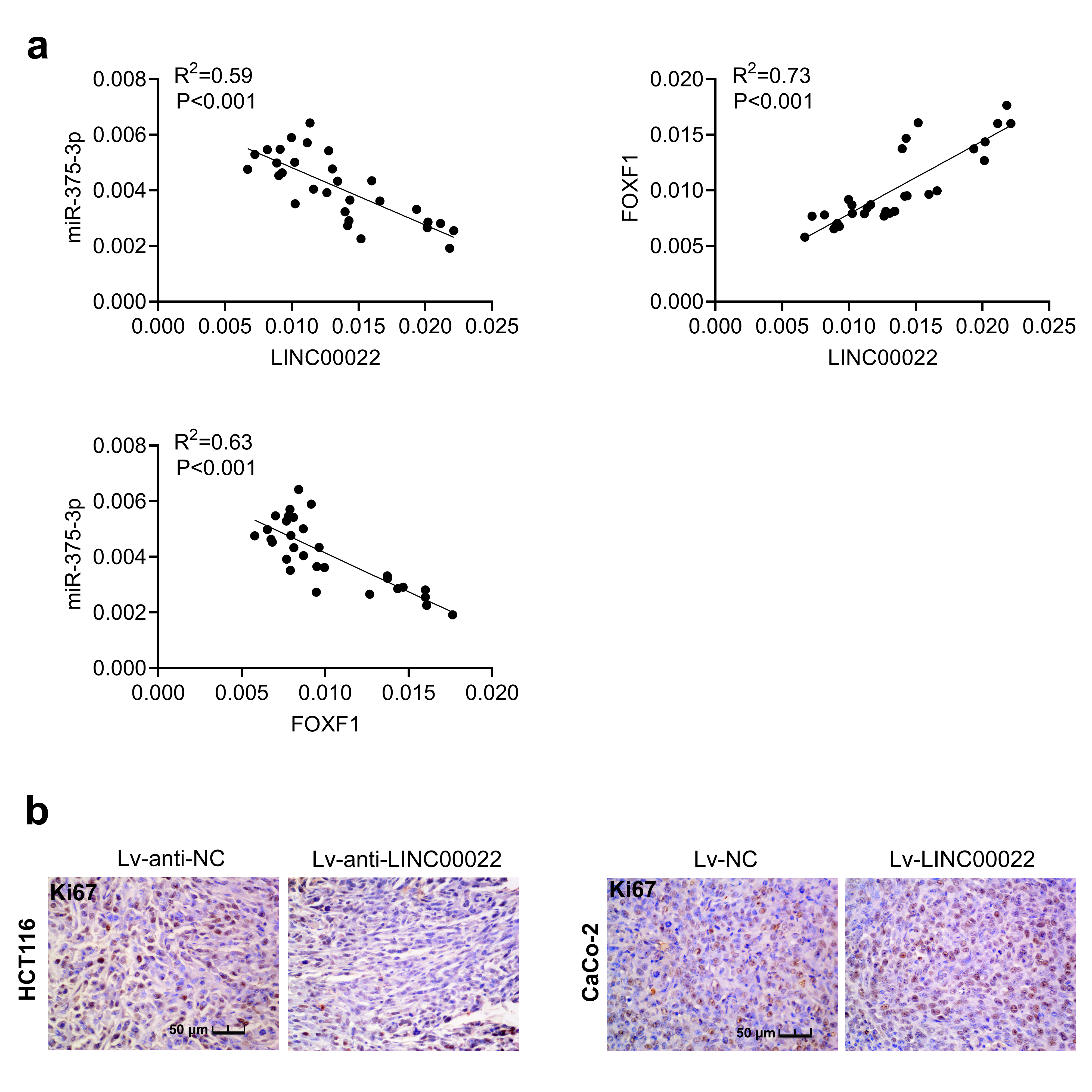

Supplement: Supplementary file 1 — Additional file 1: Supplementary figure 1. (a) The expression correlation betweenLLINC0022 and miR-375-3p, FOXP1 in CRC tissues was analyzed by quantitativereal-time PCR. (b) Immunohistochemical staining of Ki67 in tumor tissues. Scale bar = 50 μm. [file 12885_2022_9566_MOESM1_ESM.tif]

## HCT116

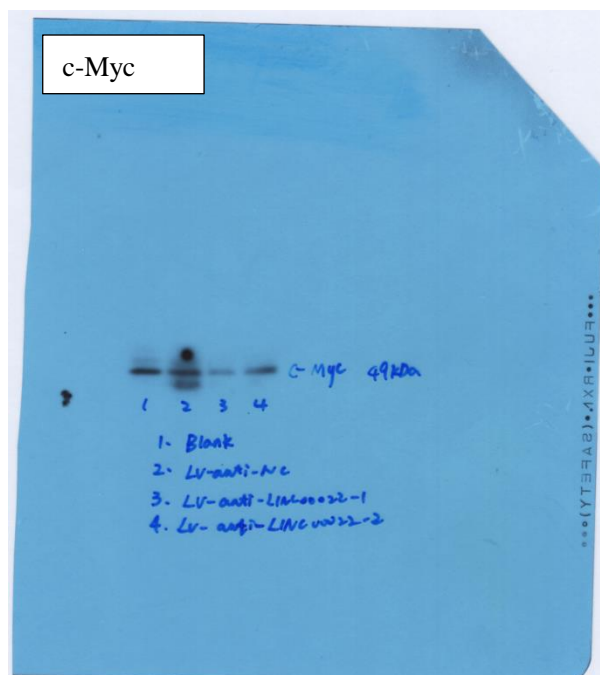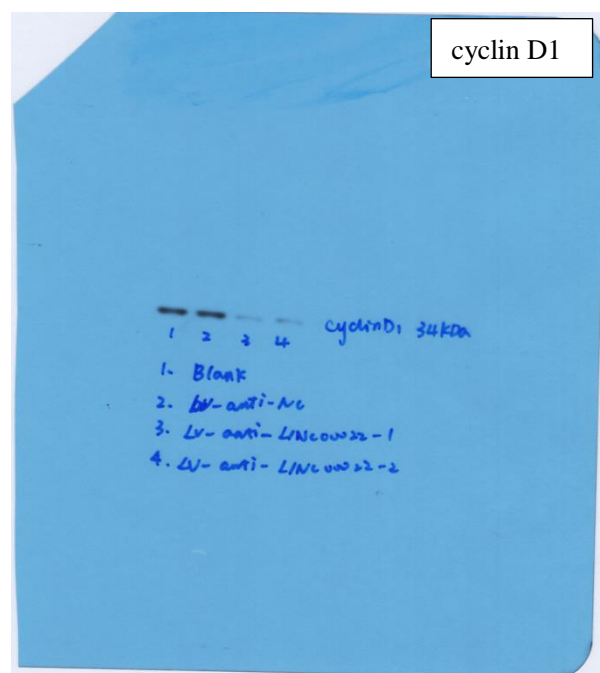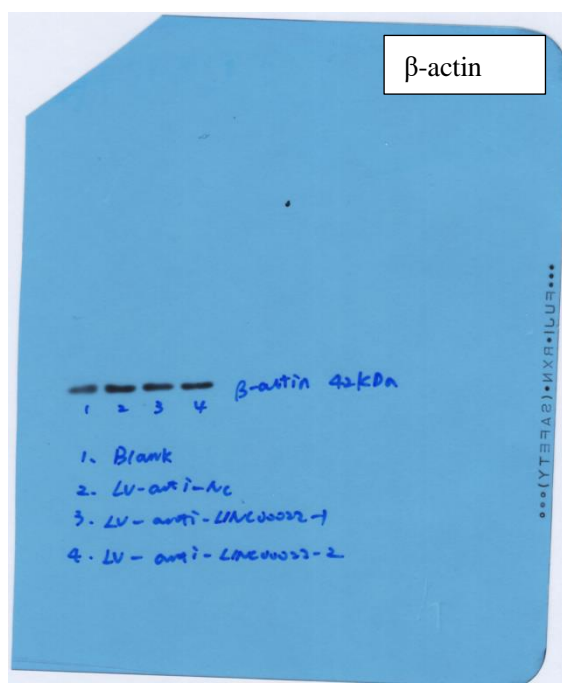

## DLD1

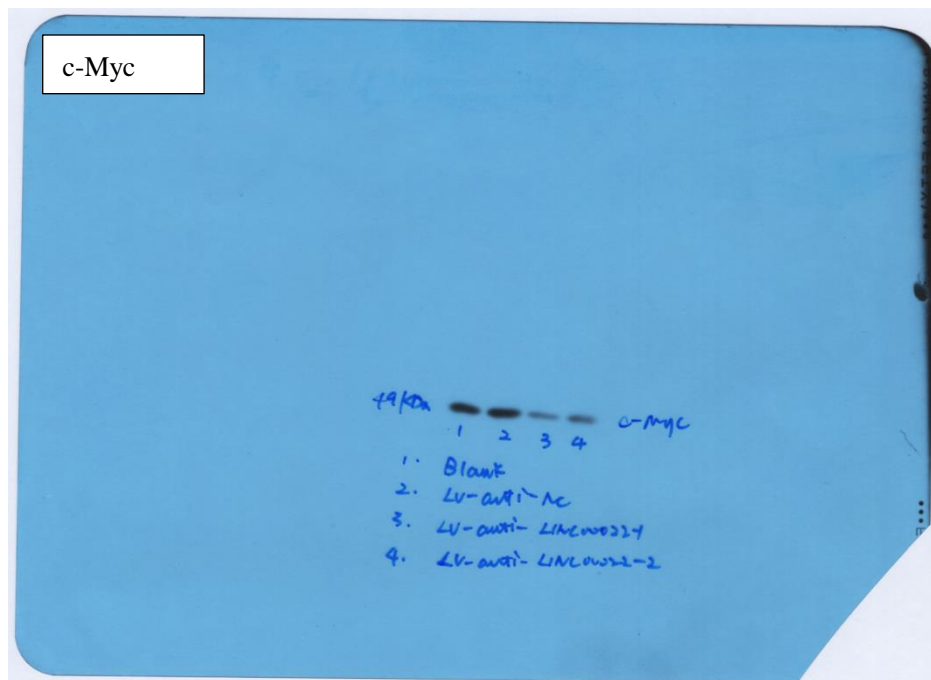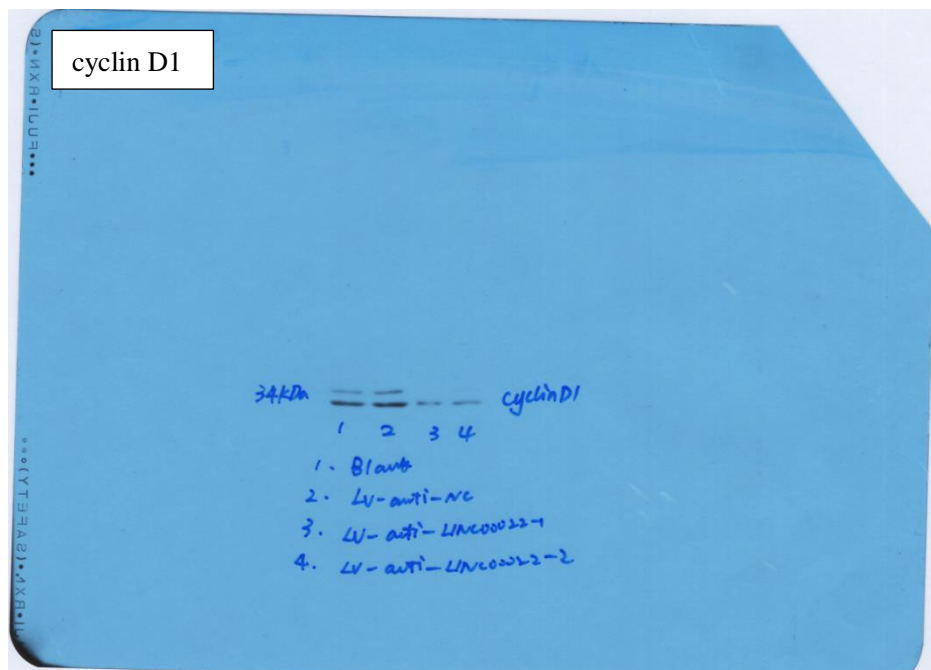

$\beta$ -actin

42kDa 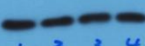  $\beta$ -actin

1 2 3 4

1. Blank
2. LV-anti-MC
3. LV-anti-LIN28022-1
4. LV-anti-LIN28022-2

••EQUJ•RXN•(SAFETY)•••

## CaCo-2

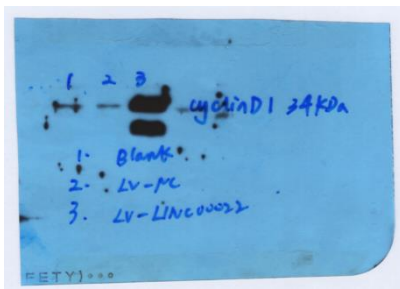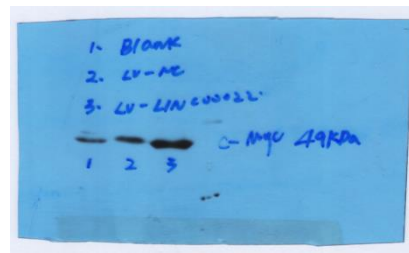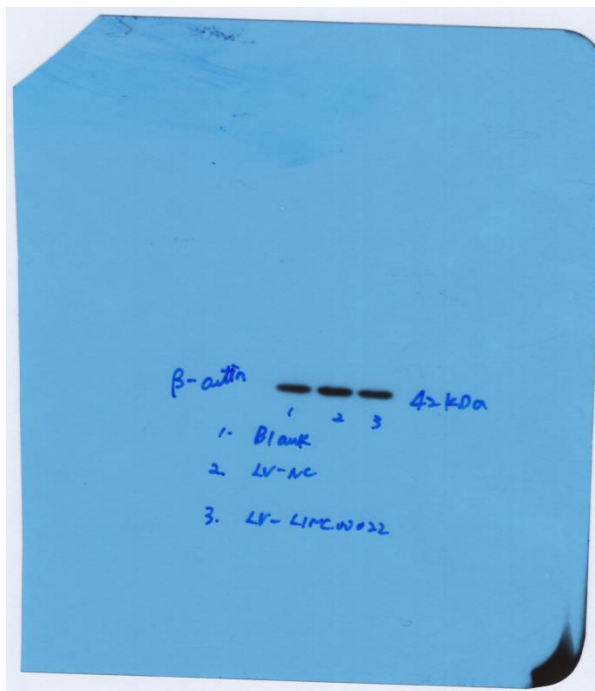

Supplement: Supplementary file 2 — Additional file 2: Supplementary figure S2d. The original blot images of Fig. 2d. [file 12885_2022_9566_MOESM2_ESM.pdf]

Supplementary Fig. S5c  
The original blot images of Fig. 5c.

## HCT116

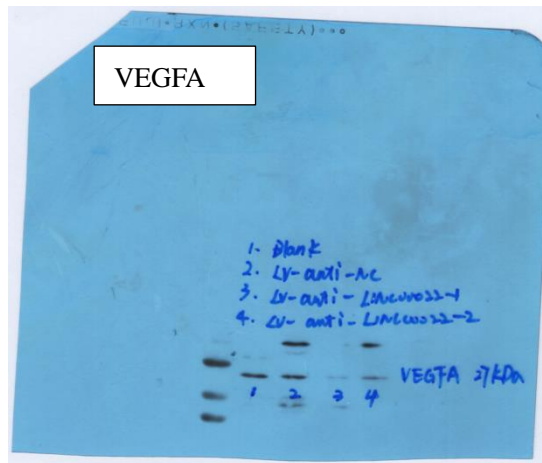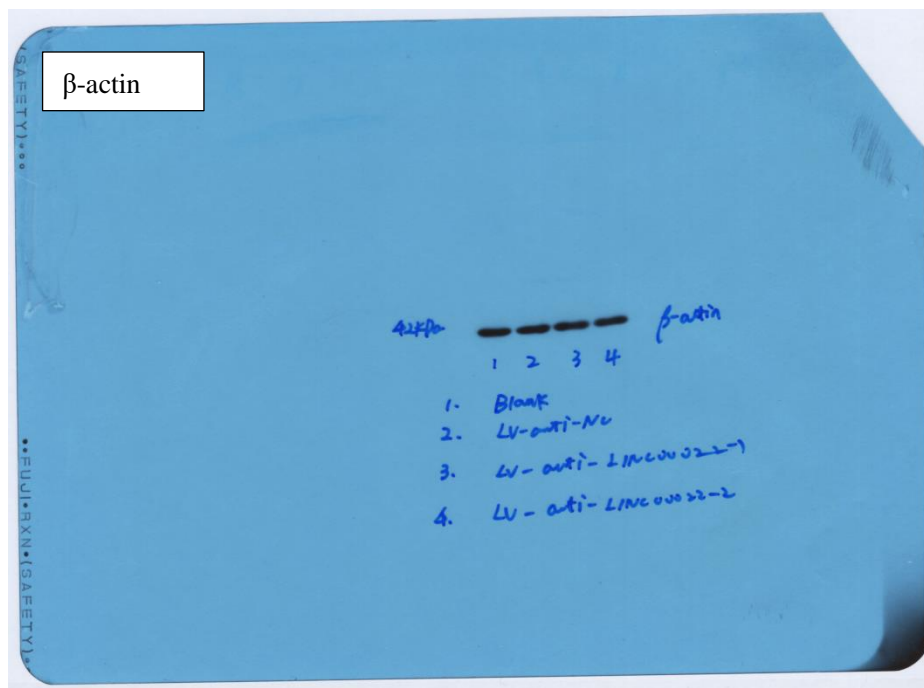

## DLD1

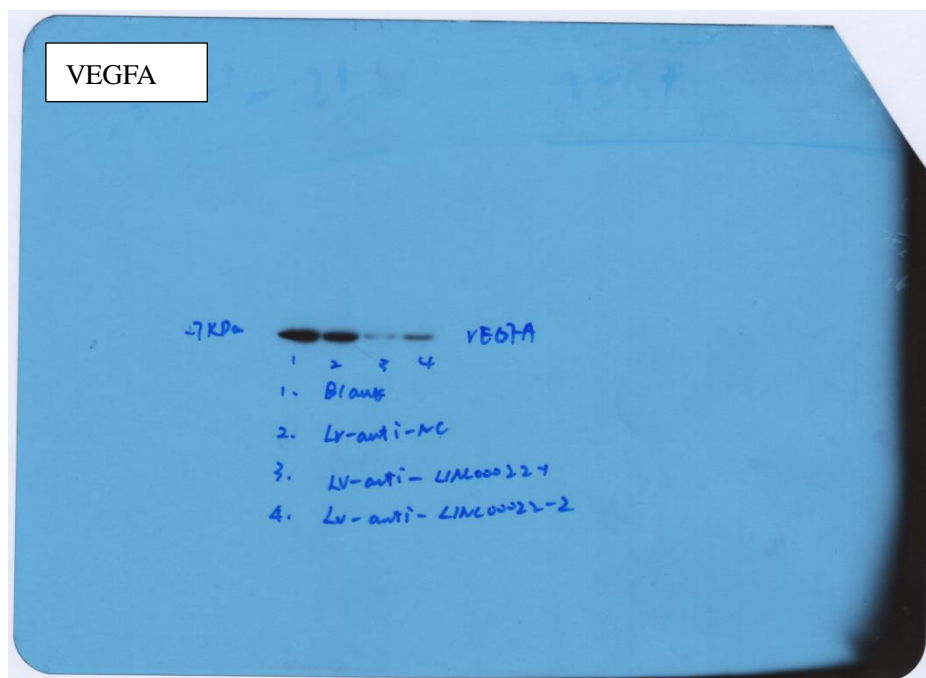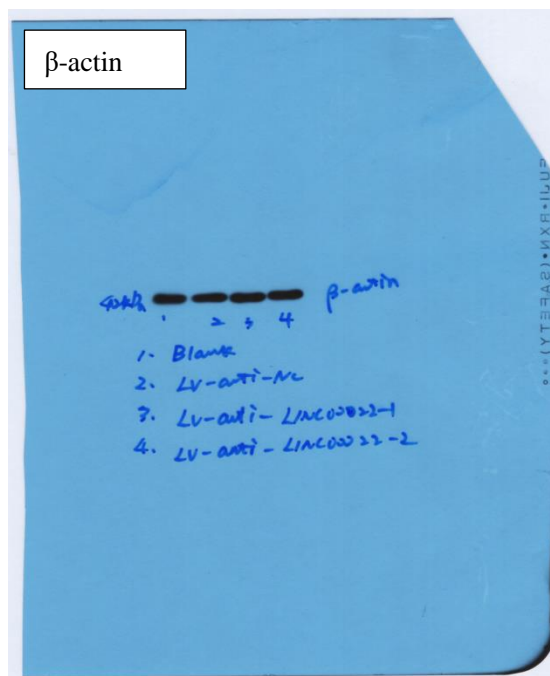

## Coco-2

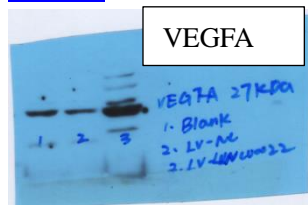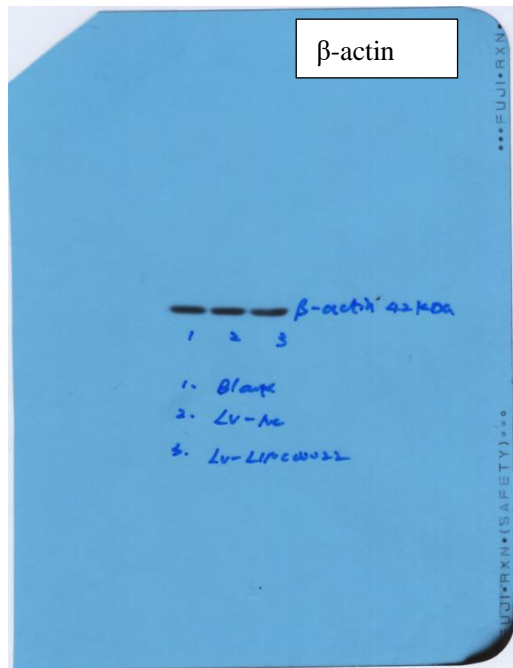

Supplement: Supplementary file 5 — Additional file 5: Supplementary figure S5c. The original blot images of Fig. 5c. [file 12885_2022_9566_MOESM5_ESM.pdf]

Supplementary Fig. S8e  
The original blot images of Fig. 8e.

**HCT116**

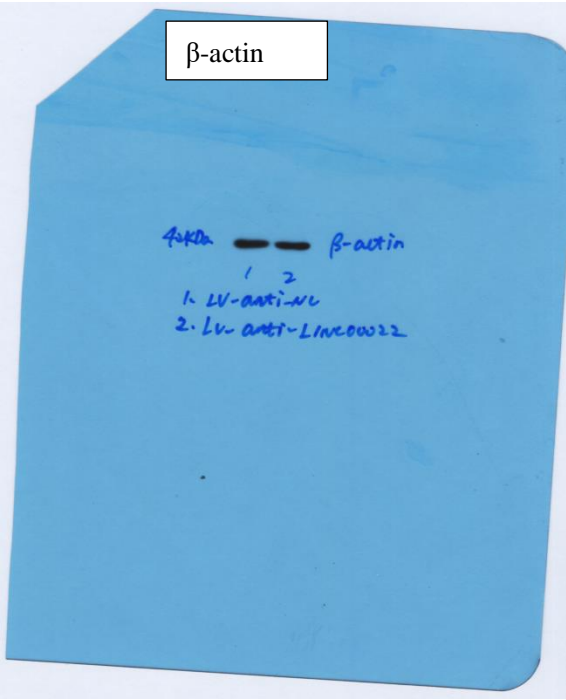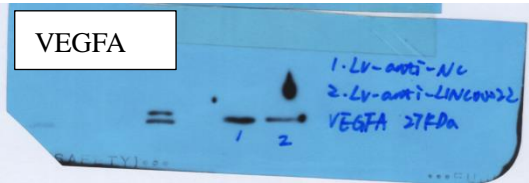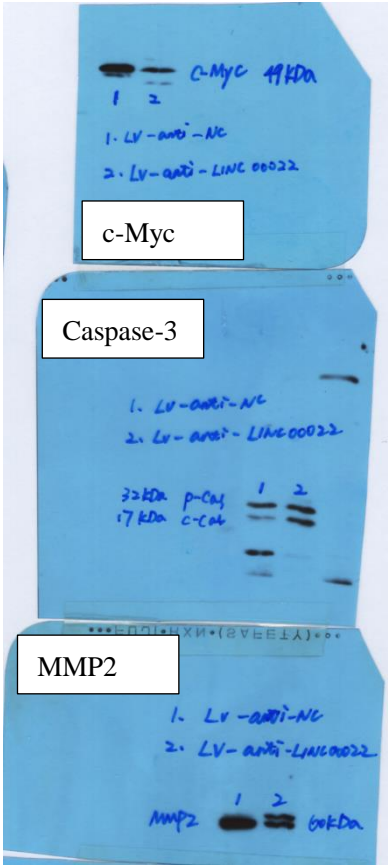

## CaCo-2

VEGFA

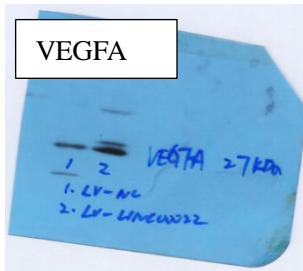

c-Myc

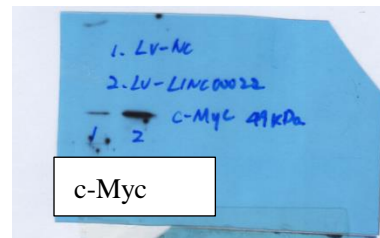

$\beta$ -actin

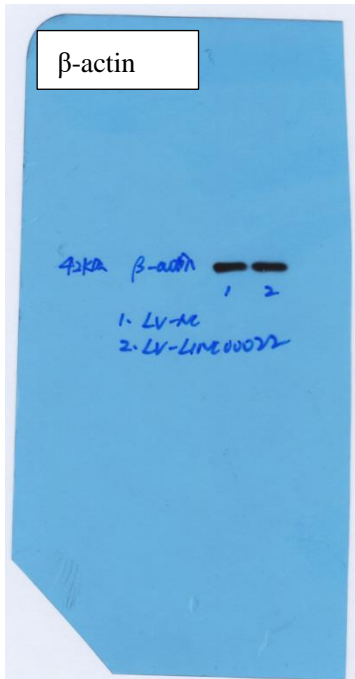

MMP2

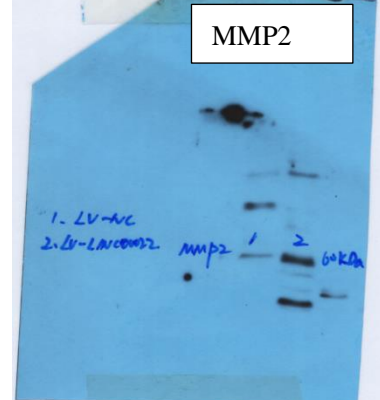

Supplement: Supplementary file 8 — Additional file 8: Supplementary figure S8e. The original blot images of Fig. 8e. [file 12885_2022_9566_MOESM8_ESM.pdf]
