# Supplementary material for: LINC00022 acts as an oncogene in colorectal cancer progression via sponging miR-375-3p to regulate FOXF1 expression
Source: BMC Cancer. 2022 Apr 26;22:453. doi: 10.1186/s12885-022-09566-5 (PMC9040237; doi:10.1186/s12885-022-09566-5)

Supplementary Fig. S3b
The original blot images of Fig. 3b.

HCT116

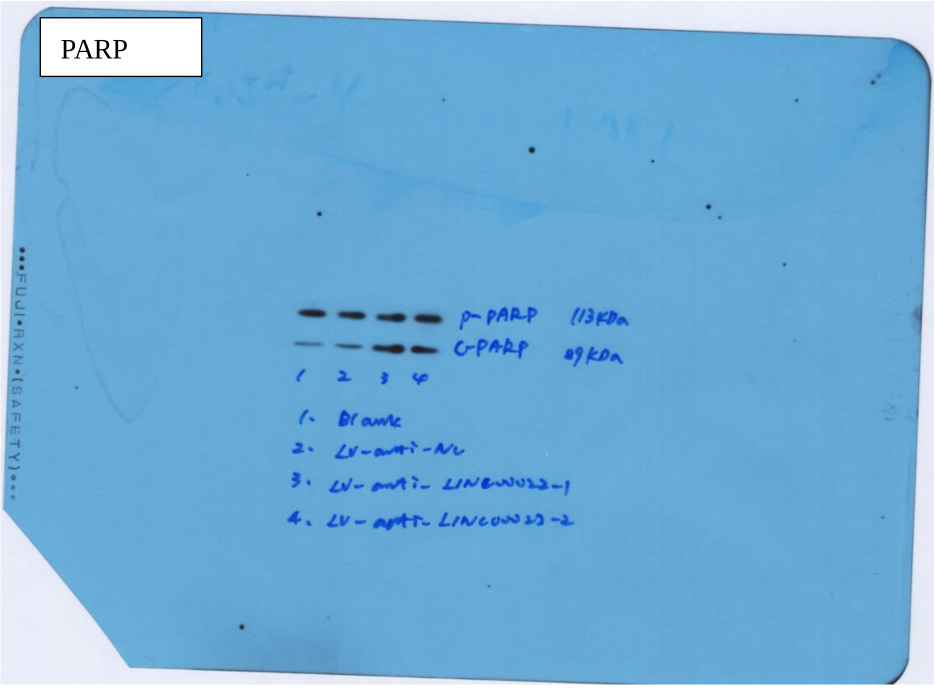

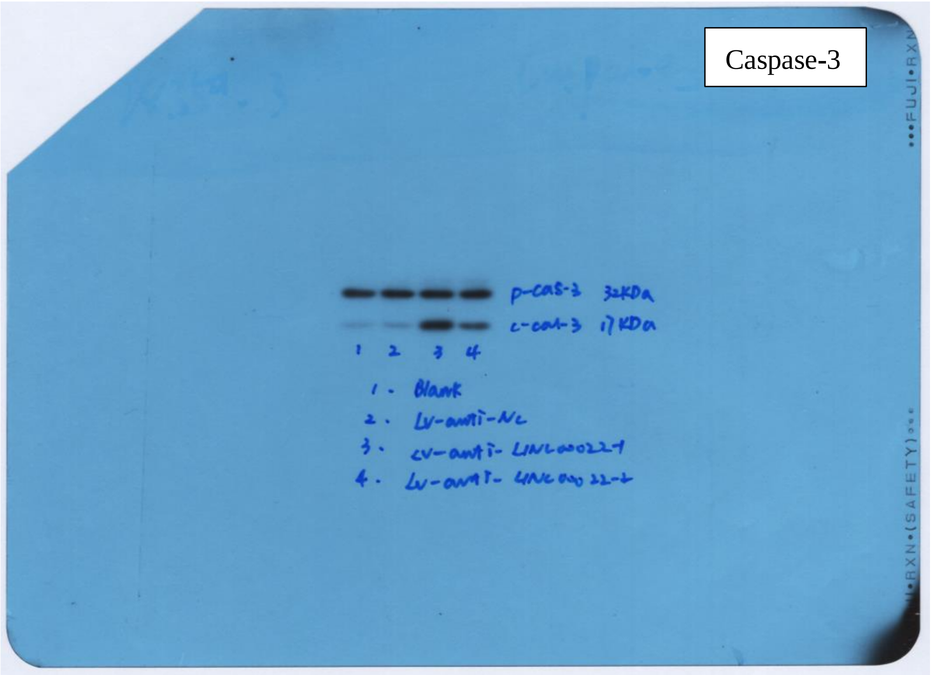

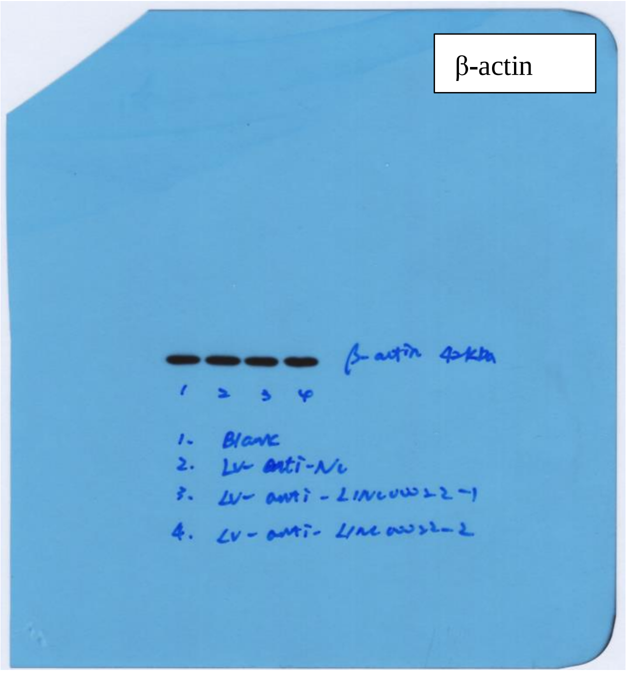
β-actin
β-actin 42kDa
1 2 3 4
1. Blank
2. LV-anti-NC

DLD1

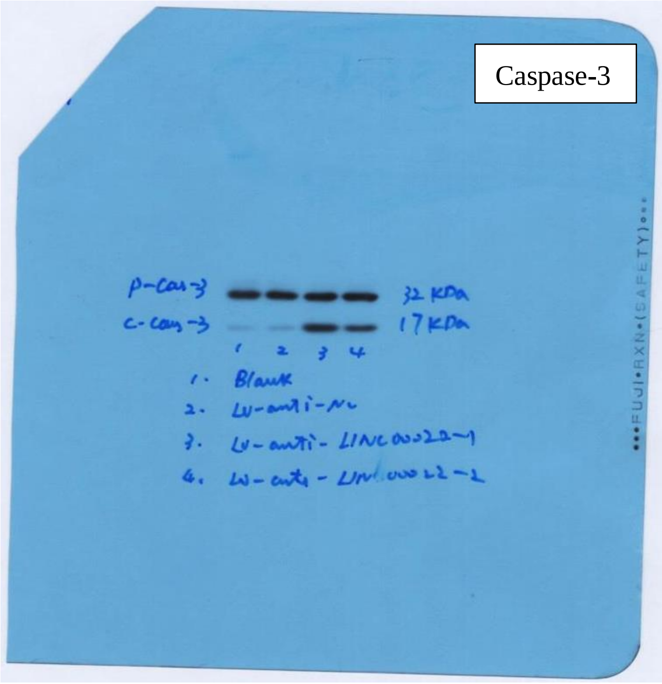

Caspase-3
p-Cas-3
c-Cas-3
32 KDa
17 KDa
1 2 3 4
1. Blank
2. LV-anti-NC
3. LV-anti-LINC00022-1
4. LV-anti-LINC00022-2

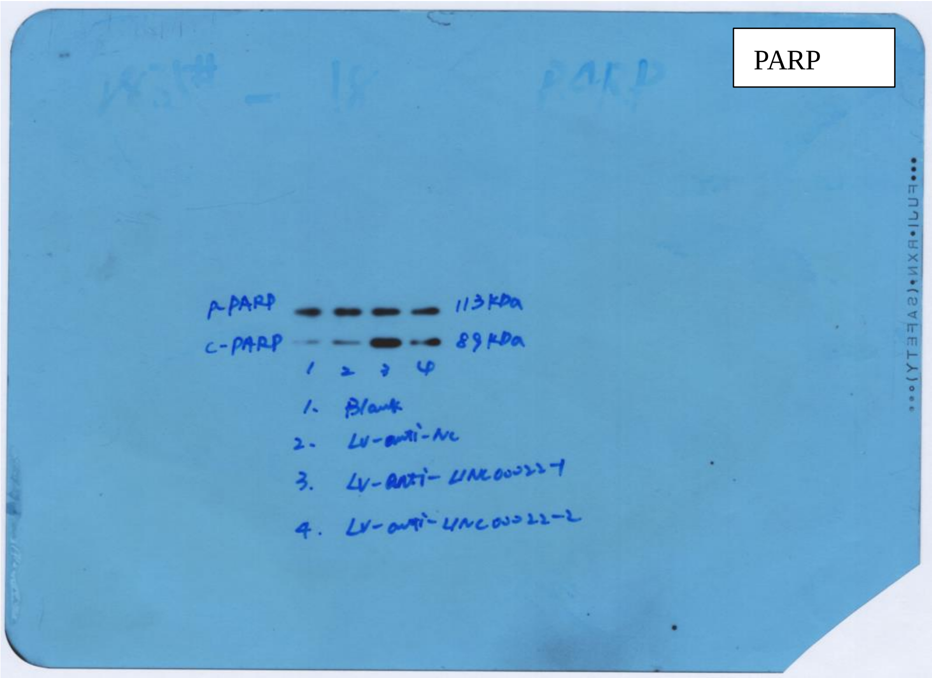

PARP
p-PARP
c-PARP
113 KDa
89 KDa
1 2 3 4
1. Blank
2. LV-anti-NC
3. LV-anti-LINC00022-1
4. LV-anti-LINC00022-2

β-actin

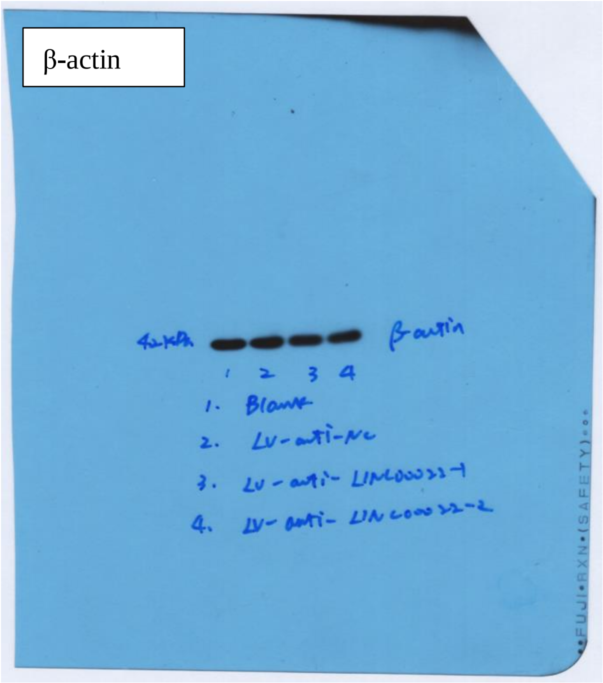

Supplement: Supplementary file 3 — Additional file 3: Supplementary figure S3b. The original blot images of Fig. 3b. [file 12885_2022_9566_MOESM3_ESM.pdf]
