# Supplementary material for: LINC00022 acts as an oncogene in colorectal cancer progression via sponging miR-375-3p to regulate FOXF1 expression
Source: BMC Cancer. 2022 Apr 26;22:453. doi: 10.1186/s12885-022-09566-5 (PMC9040237; doi:10.1186/s12885-022-09566-5)
Supplement: Supplementary file 4 — Additional file 4: Supplementary figure S4c. The original blot images of Fig. 4c. [file 12885_2022_9566_MOESM4_ESM.pdf]

Supplementary Fig. S4c
The original blot images of Fig. 4c.

HCT116

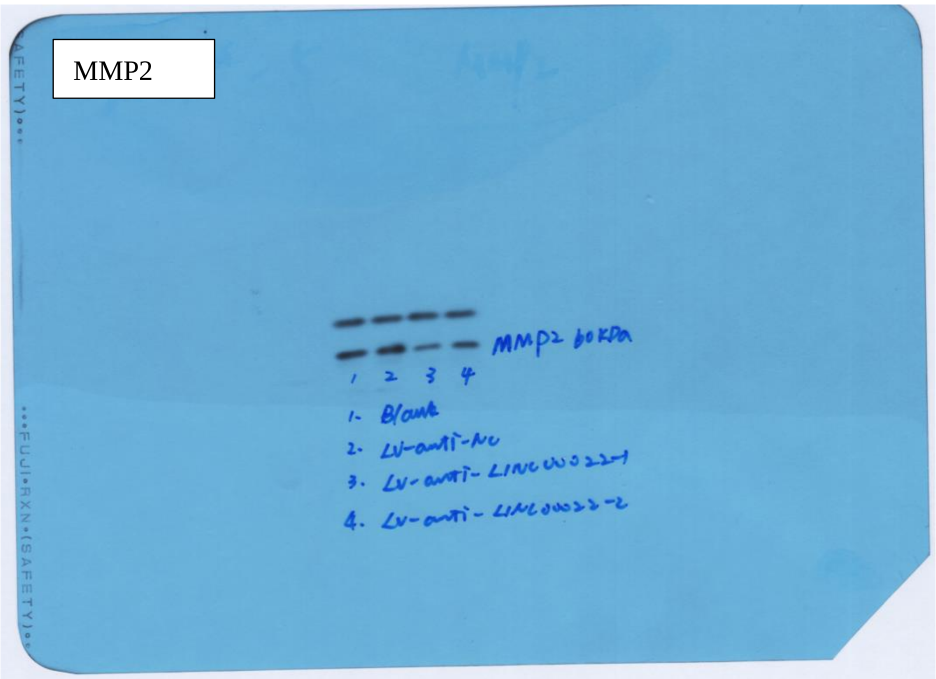

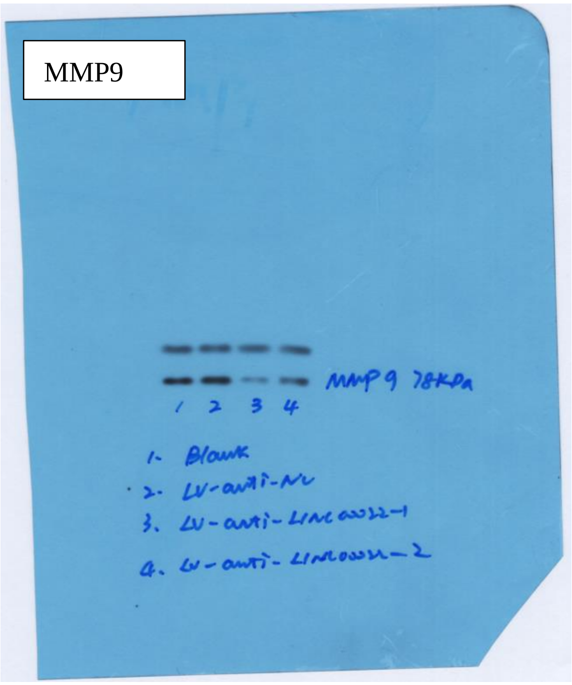

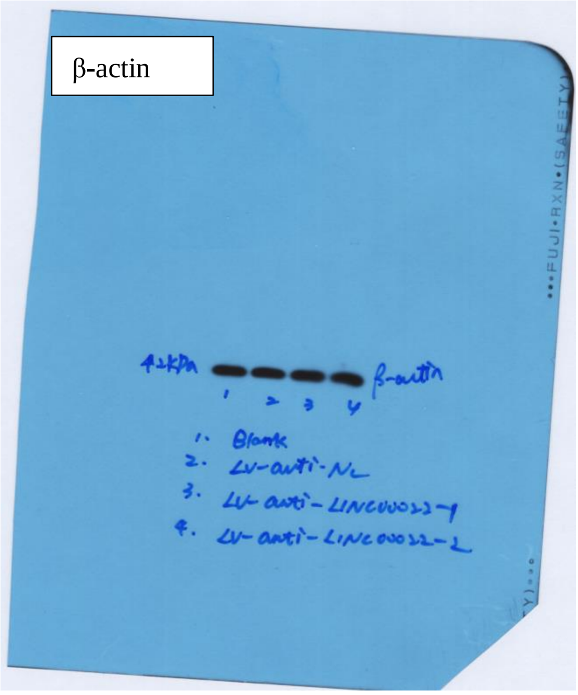

DLD1

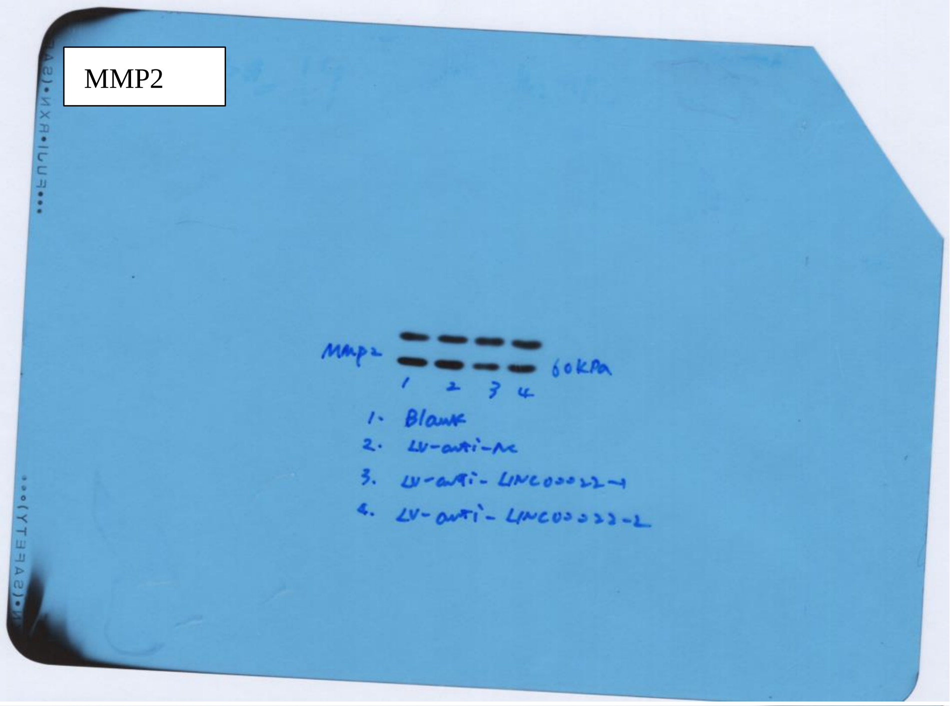

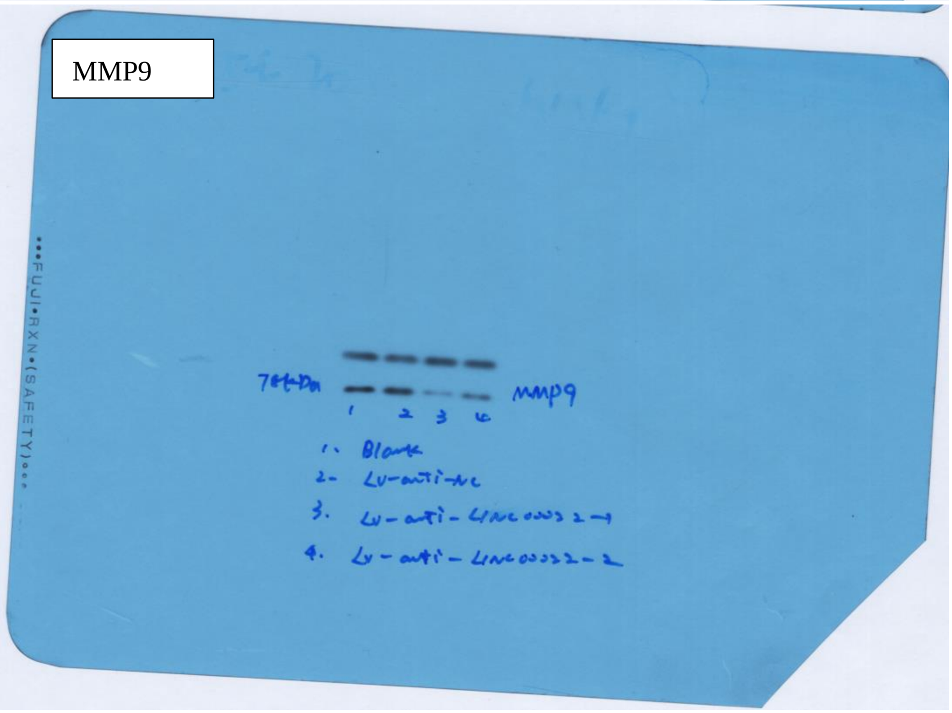

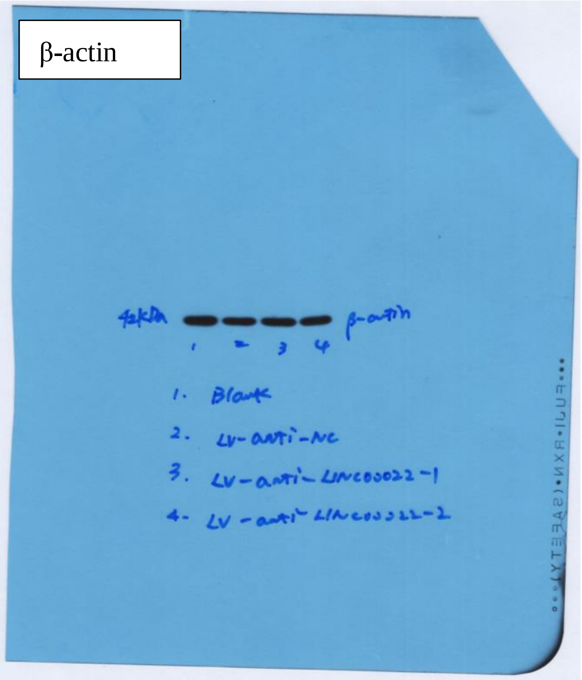
β-actin
42kDa
β-actin
1
2
3
4
1. Blank
2. LV-anti-NC
3. LV-anti-LINC00022-1
4. LV-anti-LINC00022-2

Caco-2

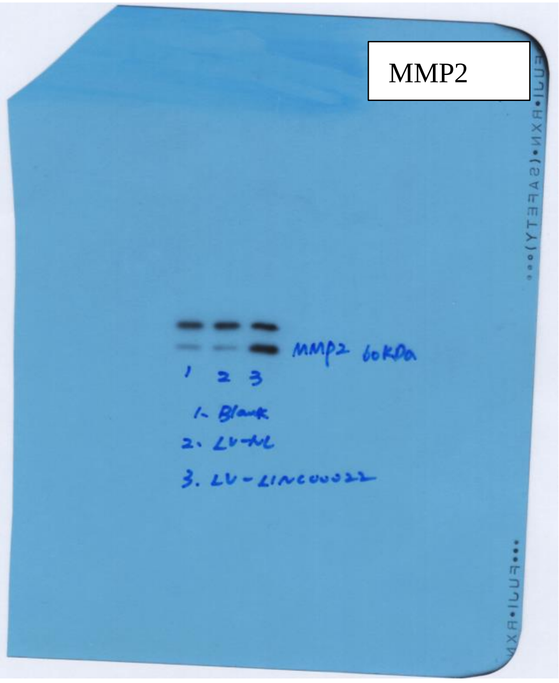
MMP2
MMP2 60kDa
1 2 3
1. Blank
2. LV-NC
3. LV-LINC00022

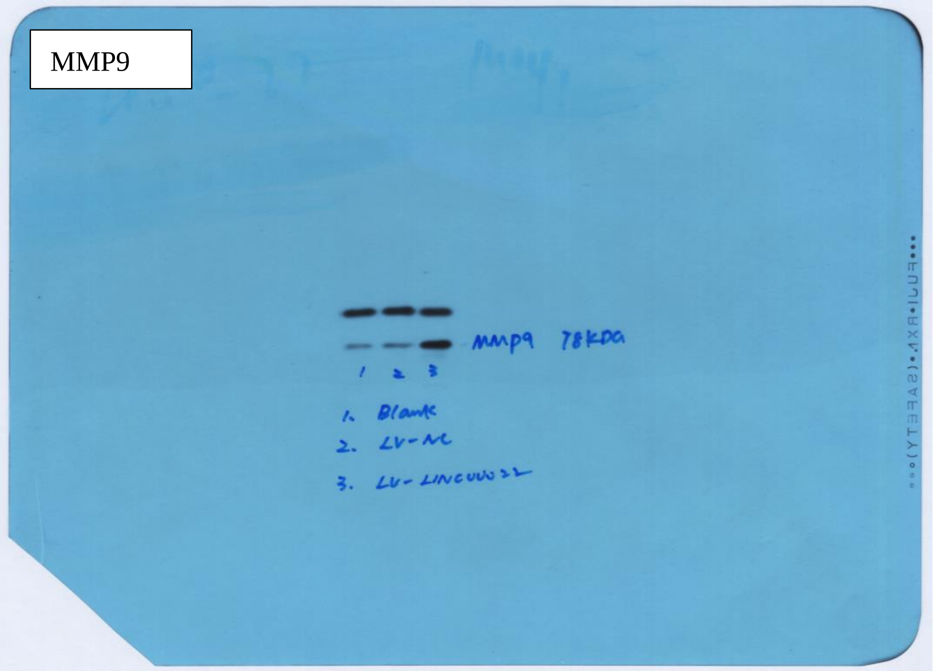
MMP9
MMP9 78kDa
1 2 3
1. Blank
2. LV-NC
3. LV-LINC00022

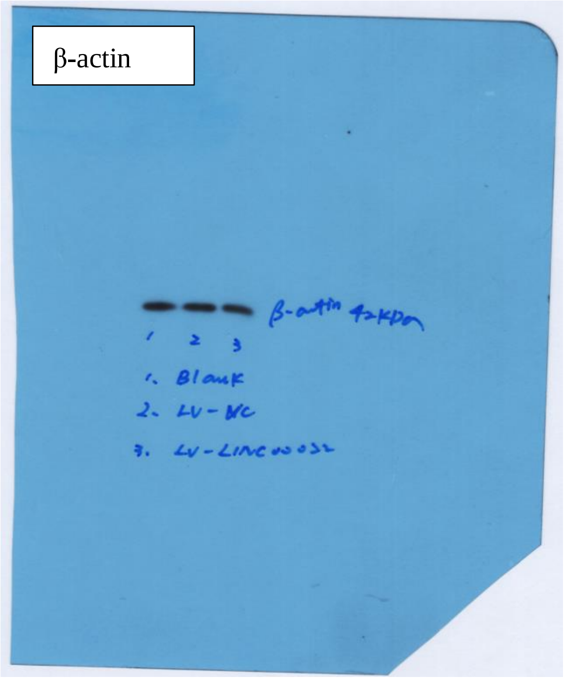
β-actin
β-actin 42KDa
1 2 3
1. Blank
2. LV-NC
3. LV-LINC00032
