# Supplementary material for: LINC00022 acts as an oncogene in colorectal cancer progression via sponging miR-375-3p to regulate FOXF1 expression
Source: BMC Cancer. 2022 Apr 26;22:453. doi: 10.1186/s12885-022-09566-5 (PMC9040237; doi:10.1186/s12885-022-09566-5)

Supplementary Fig. S6c
The original blot images of Fig. 6c.

HCT116

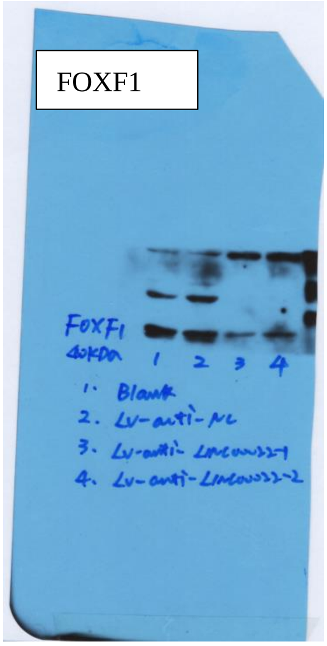

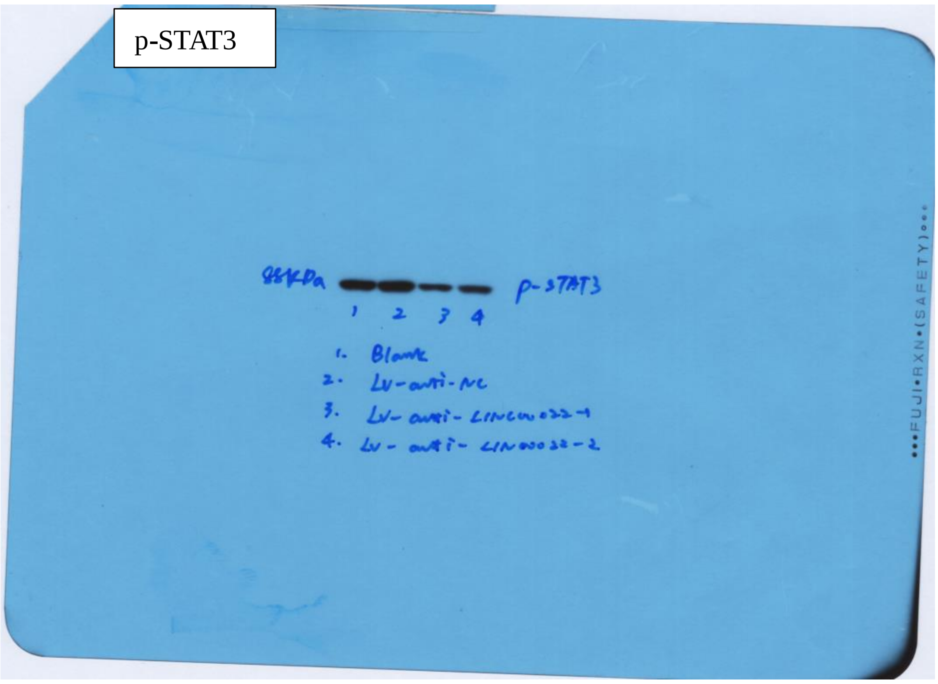

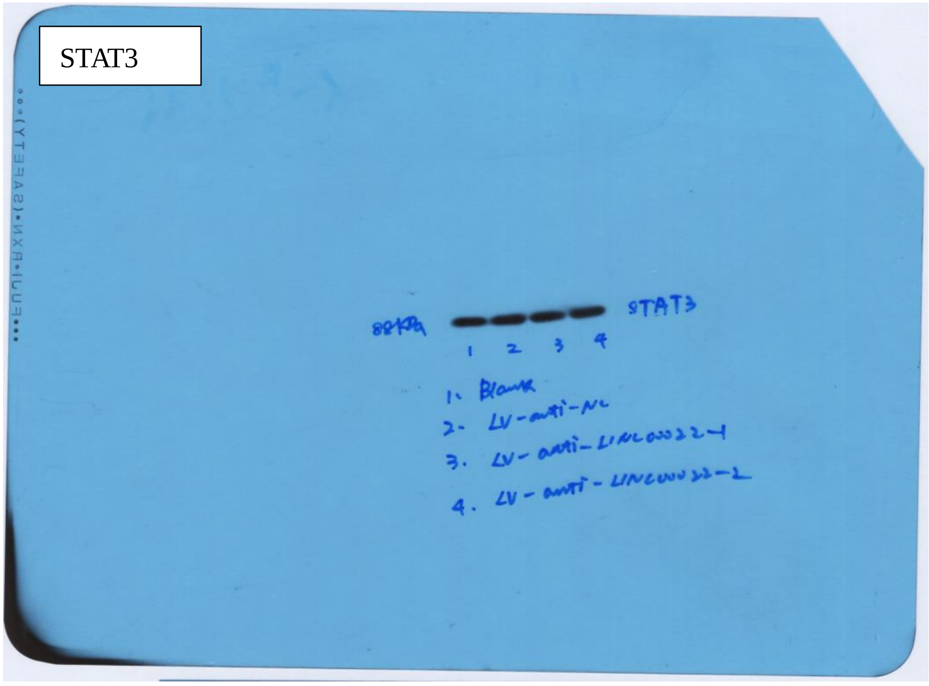

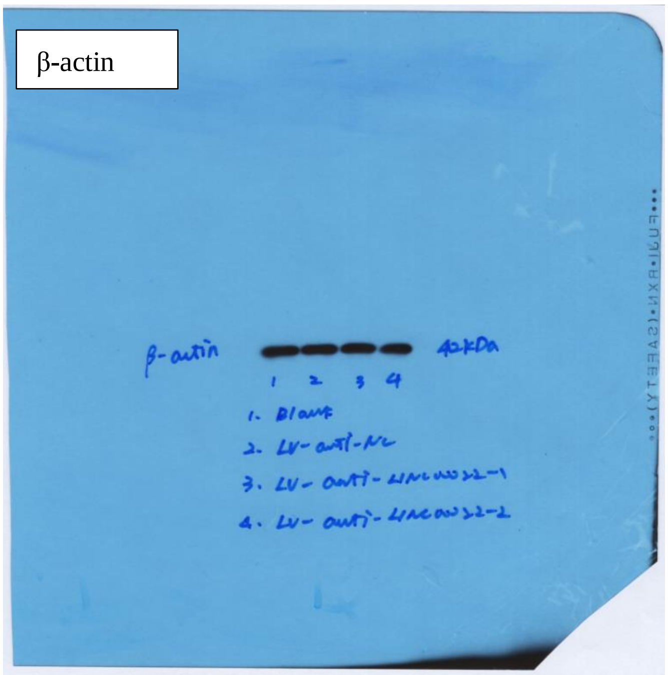
β-actin
β-actin
42kDa
1 2 3 4
1. Blank
2. LV-anti-NC
3. LV-anti-LINC00312-1
4. LV-anti-LINC00312-2

DLD1

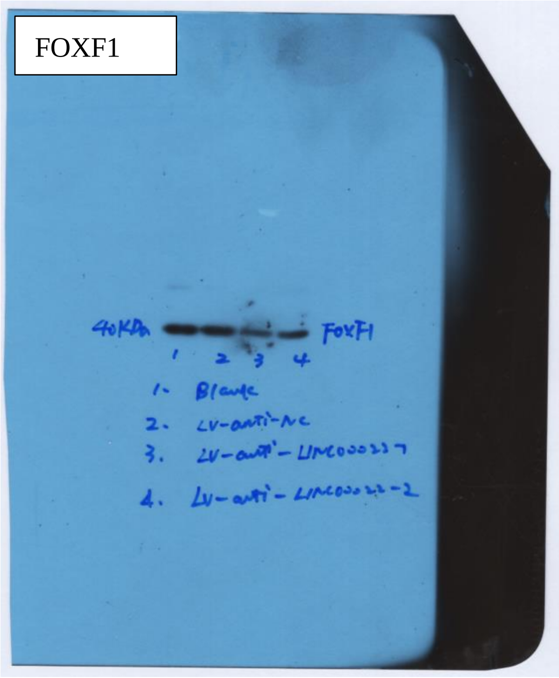

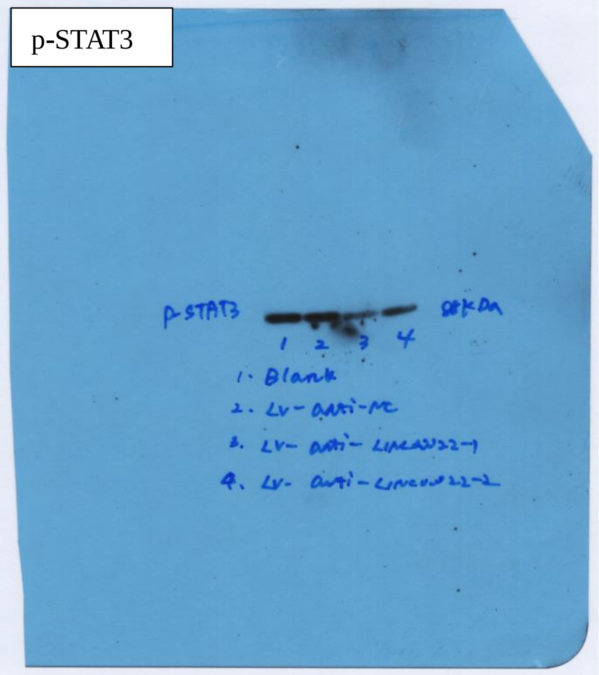

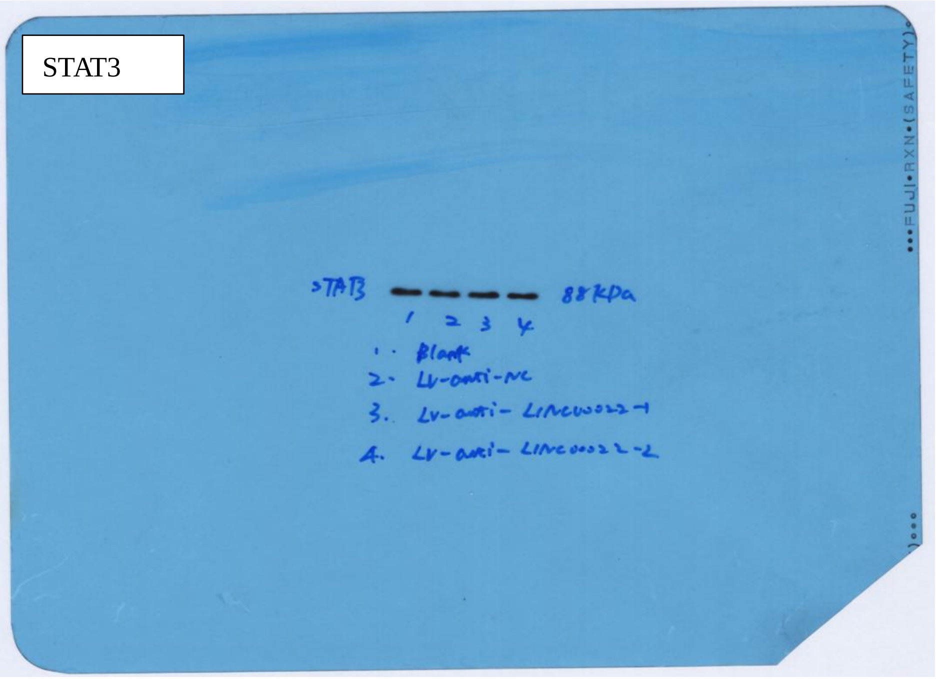

β-actin

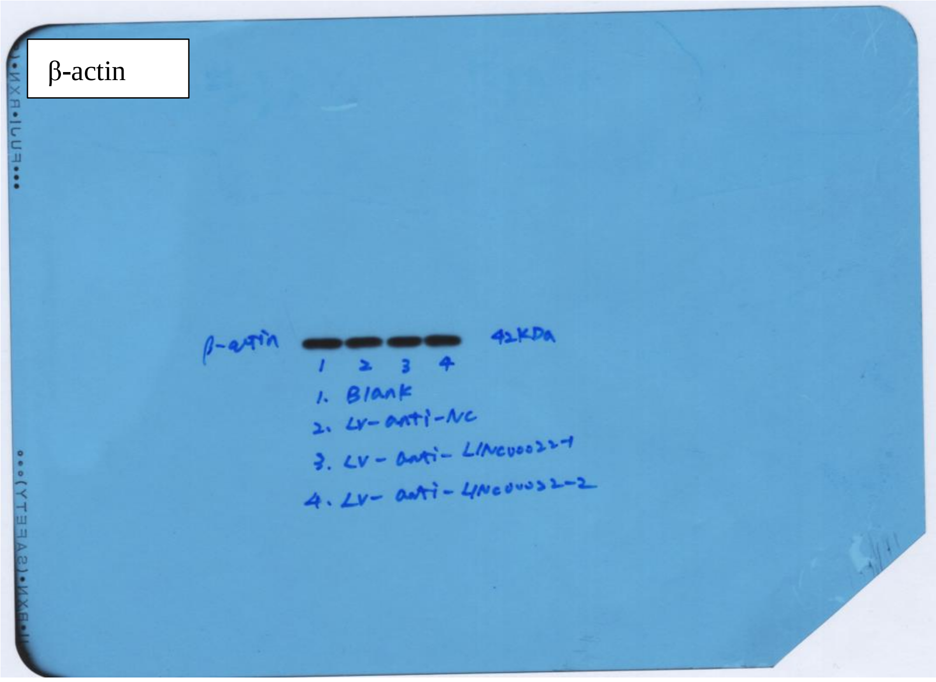

CaCo-2

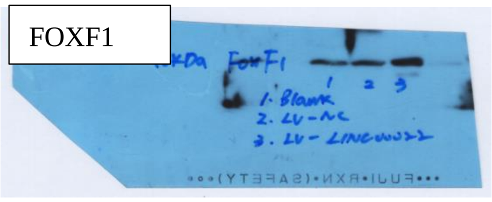

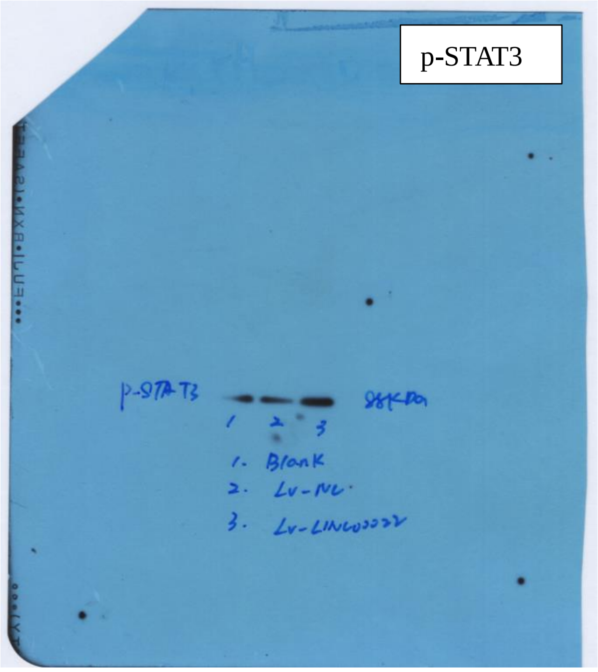

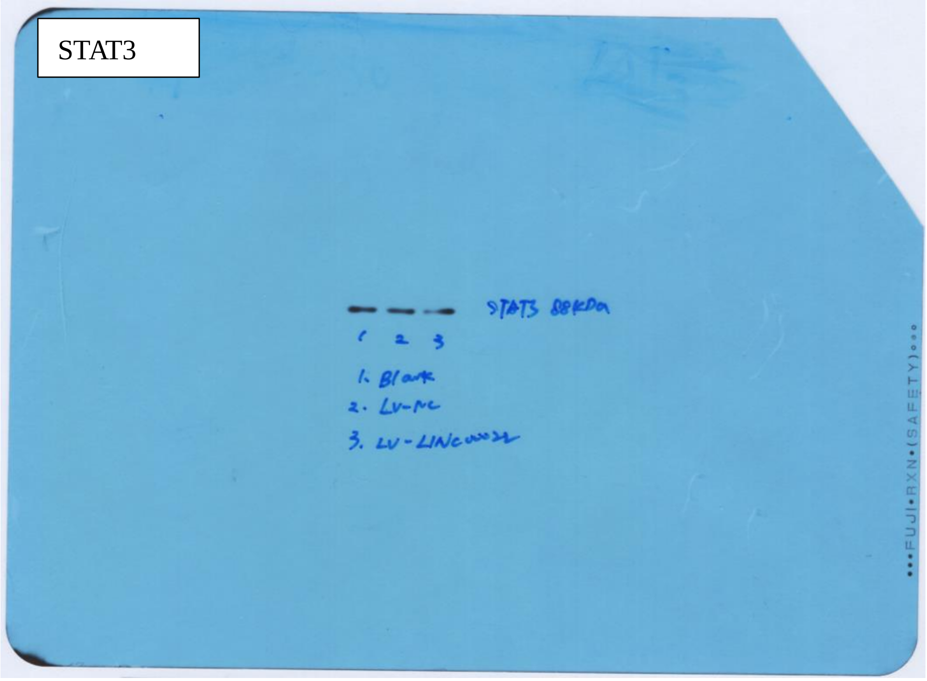

β-actin

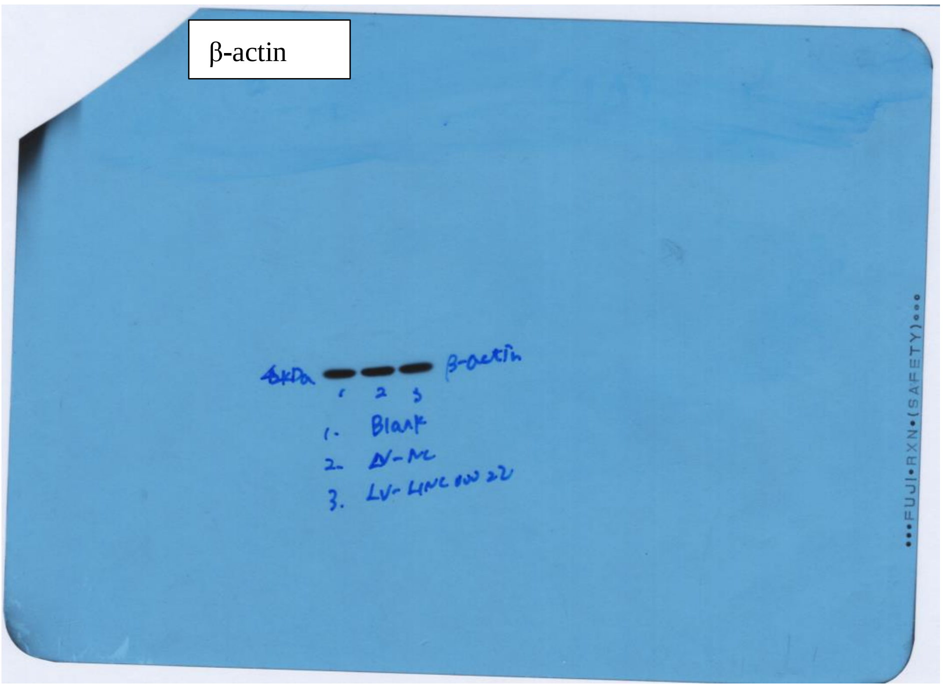

Supplement: Supplementary file 6 — Additional file 6: Supplementary figure S6c. The original blot images of Fig. 6c. [file 12885_2022_9566_MOESM6_ESM.pdf]
