# Supplementary material for: LINC00022 acts as an oncogene in colorectal cancer progression via sponging miR-375-3p to regulate FOXF1 expression
Source: BMC Cancer. 2022 Apr 26;22:453. doi: 10.1186/s12885-022-09566-5 (PMC9040237; doi:10.1186/s12885-022-09566-5)
Supplement: Supplementary file 7 — Additional file 7: Supplementary figure S7d. The original blot images of Fig. 7d. [file 12885_2022_9566_MOESM7_ESM.pdf]

Supplementary Fig. S7d
The original blot images of Fig. 7d.

HCT116

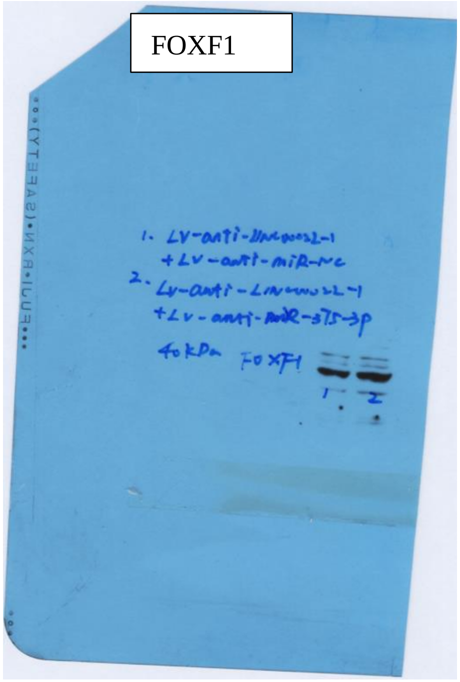

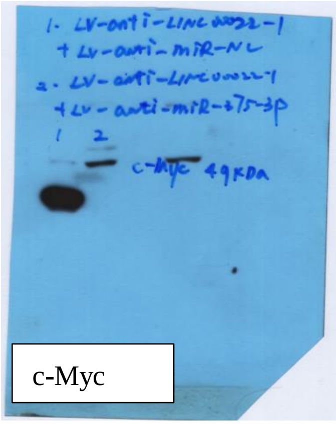

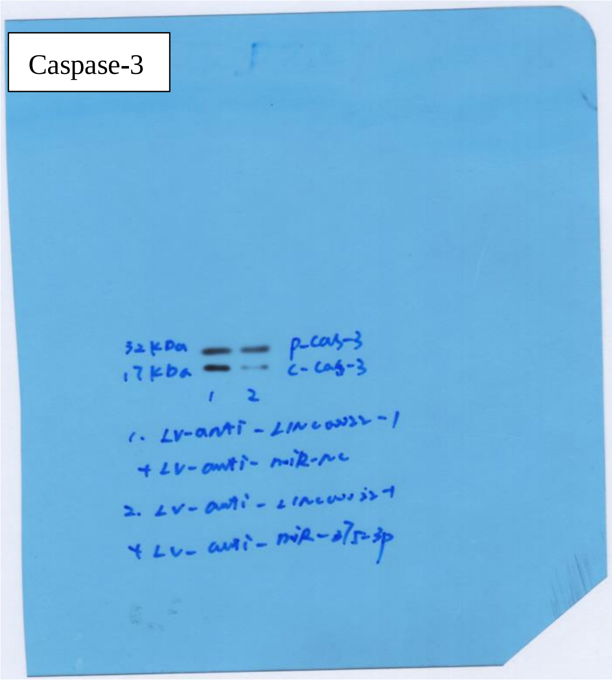

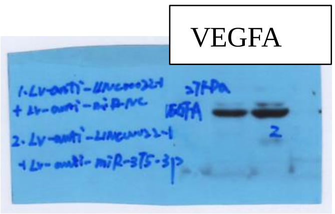

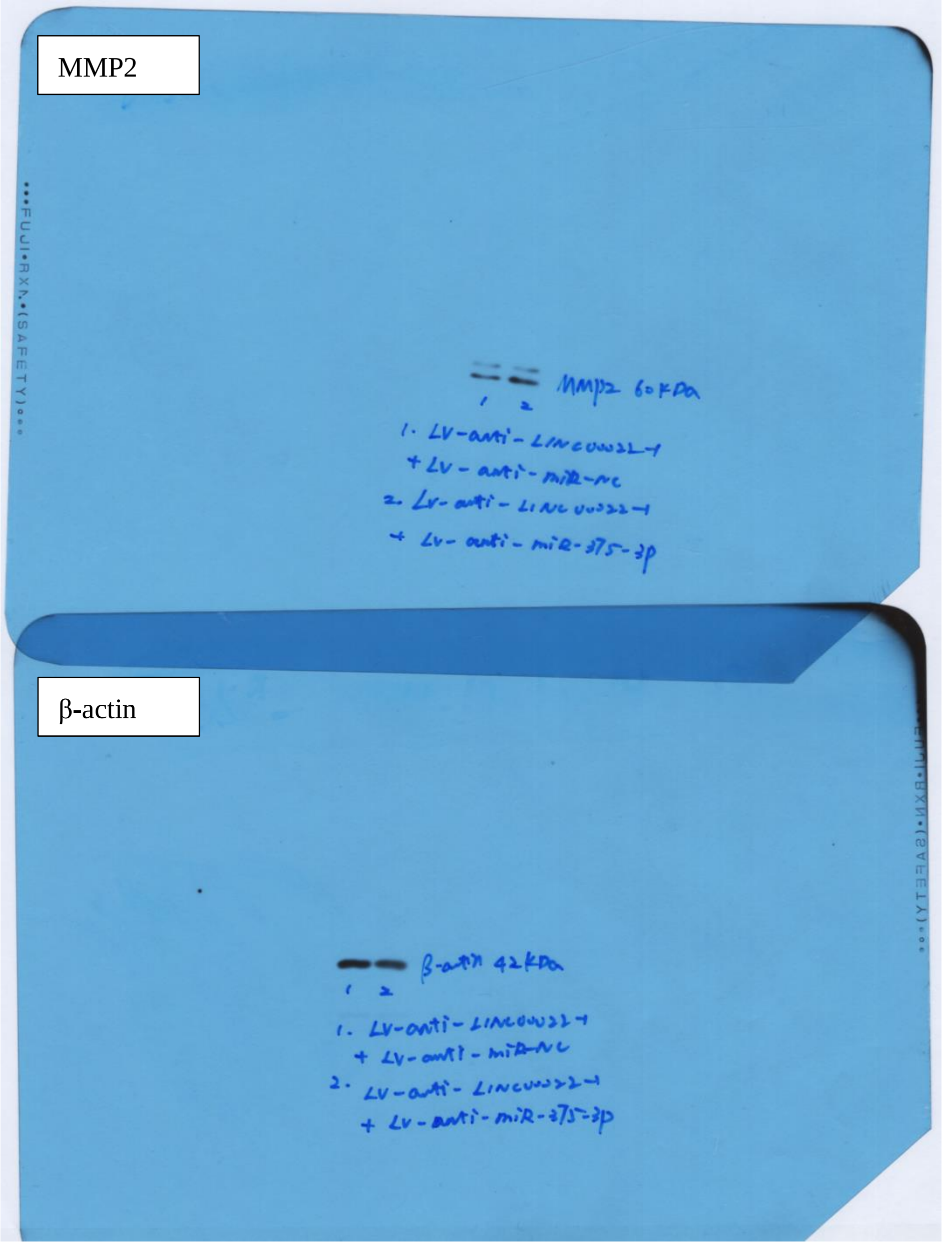
MMP2
MMP2 60kDa
β-actin
β-actin 42kDa
